# Isolation and comparative proteomic analysis of mitochondria from the pulp of ripening citrus fruit

**DOI:** 10.1038/s41438-021-00470-w

**Published:** 2021-02-01

**Authors:** Xin Li, Yingfang Chai, Hongbin Yang, Zhen Tian, Chengyang Li, Rangwei Xu, Chunmei Shi, Feng Zhu, Yunliu Zeng, Xiuxin Deng, Pengwei Wang, Yunjiang Cheng

**Affiliations:** National R&D Centre for Citrus Preservation, Key Laboratory of Horticultural Plant Biology (Ministry of Education), College of Horticulture and Forestry Science, Huazhong Agricultural University, Wuhan, 430070 People’s Republic of China

**Keywords:** Plant sciences, Cell biology

## Abstract

Mitochondria are crucial for the production of primary and secondary metabolites, which largely determine the quality of fruit. However, a method for isolating high-quality mitochondria is currently not available in citrus fruit, preventing high-throughput characterization of mitochondrial functions. Here, based on differential and discontinuous Percoll density gradient centrifugation, we devised a universal protocol for isolating mitochondria from the pulp of four major citrus species, including satsuma mandarin, ponkan mandarin, sweet orange, and pummelo. Western blot analysis and microscopy confirmed the high purity and intactness of the isolated mitochondria. By using this protocol coupled with a label-free proteomic approach, a total of 3353 nonredundant proteins were identified. Comparison of the four mitochondrial proteomes revealed that the proteins commonly detected in all proteomes participate in several typical metabolic pathways (such as tricarboxylic acid cycle, pyruvate metabolism, and oxidative phosphorylation) and pathways closely related to fruit quality (such as γ-aminobutyric acid (GABA) shunt, ascorbate metabolism, and biosynthesis of secondary metabolites). In addition, differentially abundant proteins (DAPs) between different types of species were also identified; these were found to be mainly involved in fatty acid and amino acid metabolism and were further confirmed to be localized to the mitochondria by subcellular localization analysis. In summary, the proposed protocol for the isolation of highly pure mitochondria from different citrus fruits may be used to obtain high-coverage mitochondrial proteomes, which can help to establish the association between mitochondrial metabolism and fruit storability or quality characteristics of different species and lay the foundation for discovering novel functions of mitochondria in plants.

## Introduction

Mitochondria, whose origin is described by the universally acknowledged endosymbiosis theory^1^, play a central role in eukaryotic cells by providing energy in the form of ATP via aerobic respiration^2^. In fleshy fruits, mitochondria are the major sites for the metabolism of organic acids, amino acids, and lipids as well as the biosynthesis of vitamins and other cofactors, and they are involved in the development and maintenance of fruit quality^3,4^. Mitochondria are characterized by an elaborate structure consisting of four subcompartments, namely, the inner and outer membranes, the intermembrane space, and the matrix. Multiple metabolic reactions and biochemical processes take place within this relatively small compartment in a well-coordinated way^5^. Thus, membrane integrity is essential for proper mitochondrial function.

Mitochondria are semiautonomous organelles with their own genomes and undergo transcription and translation^6^. It has been estimated that mitochondria synthesize less than 5% of the proteins required for their functions; the remaining 95% of required proteins are encoded by the nuclear genome, translated in the cytosol, and imported into the mitochondria. These proteins compose the mitochondrial proteome and are involved in diverse functions of mitochondria. Hence, characterization of the mitochondrial proteome will facilitate the high-throughput elucidation of its physiological and biochemical roles.

Isolation of high-purity mitochondria is a prerequisite for high-throughput characterization of mitochondrial functions. In the earliest studies (starting in the 1950s), mitochondrial pellets (so-called crude mitochondria) were obtained by means of differential centrifugation and were very likely contaminated by other organelle fractions, such as plastids and peroxisomes^7^. Later, crude mitochondria were further purified by using density gradients such as sucrose^8^ or Percoll^9^ and free-flow electrophoresis (FFE)^10^ to remove contaminants. The plant materials used for mitochondrial extraction have been extensively reported, including some nongreen tissues such as fleshy roots or tubers and etiolated seedlings (hypocotyls, cotyledons, roots, or coleoptiles), green tissues (leaves), and cell suspension cultures^11^. However, compared with model plants or field crops, whose mitochondria can be more easily isolated by differential and density gradient centrifugation^11,12^, fleshy fruits (especially the pulp) contain high levels of interfering compounds such as sugars, cellulose, pectins, organic acids, phenolics, and pigments, making the enrichment and purification of mitochondria relatively difficult^13^. Very recently, a rapid affinity purification technique was proposed to isolate mitochondria from *Arabidopsis*, which eliminates the need for laborious centrifugation steps and large amounts of starting materials required by classic mitochondrial preparation^14–16^. However, this technique has not been applied to fruits due to the difficulty of genetic transformation.

In early studies, crude mitochondria were isolated from a number of fruit species, such as avocado, apple, and tomato, for the evaluation of their metabolic activity^17–19^. The activities of several mitochondrial enzymes were also assessed in mitochondrial isolates from banana^20^, lemon^21^, and mango^22^. The introduction of density gradient centrifugation using sucrose^23^ or Percoll^24^ greatly enhanced the purity of mitochondria isolated from fruits, making it possible to characterize the mitochondrial proteomes of various fruit crops. Subsequently, proteomic analysis revealed that oxidative damage and carbonylation modification of mitochondrial proteins are widely involved in the regulation of fruit ripening and senescence^25–28^. Differentially expressed mitochondrial proteins have also been identified at different stages of fruit ripening^29^ or in the ripening-deficient mutant^4^. In addition, mitochondrial energy metabolism and reactive oxygen species (ROS) accumulation were found to participate in the fruit defense response under postharvest pathogen infection^30–32^. Therefore, characterization of the mitochondrial proteome can provide important insight into the protein expression profile in response to biotic and abiotic stresses as well as facilitate a better understanding of the molecular mechanisms underlying fruit quality maintenance and ripening processes.

Citrus is one of the most economically important fruit crops in the world and is widely grown in more than 130 countries and regions. In contrast to tomato, a model climacteric fruit that undergoes a burst of respiration and ethylene production, citrus fruit shows typical nonclimacteric characteristics during ripening, and different species differ greatly in shelf life^33,34^. As the major organic acid accumulating in the juice sac cells of citrus fruit, citric acid is mainly synthesized via the tricarboxylic acid (TCA) cycle in mitochondria, which contributes significantly to fruit quality and storage performance and ultimately determines consumer preference^35^. Vitamin C, including ascorbic acid (AsA) and dehydroascorbic acid, is one of the most important nutritional components in citrus fruit, and its biosynthesis is also tightly controlled by mitochondria^36^. Among citrus fruits, mitochondria were first isolated from lemon via differential centrifugation^37^, and a similar method was later utilized to extract mitochondria from orange fruit, resulting in the identification of 657 mitochondria-associated proteins, which highlighted the predominant role of the citric acid cycle in determining fruit quality^38^. However, mitochondria prepared by differential centrifugation are generally of low purity. Thus, it is essential to establish a protocol for obtaining high-purity mitochondria, which will facilitate the high-throughput characterization of the mitochondrial proteome and provide new insights into the relationship between mitochondrial metabolism and fruit quality maintenance or storability.

In the present study, we optimized the protocol for the isolation of high-quality mitochondria from citrus pulp. Four major citrus species, satsuma mandarin, ponkan mandarin and sweet orange, pummelo, were used to represent the two types of commercial postharvest practices for citrus, that is, practices for loose-skin (easy to peel) citrus and tight-skin (hard to peel) citrus, respectively^39^. Then, the mitochondrial proteomes were characterized with a label-free liquid chromatography and two-dimensional mass spectrometry (LC-MS/MS)-based approach, and the differentially abundant proteins (DAPs) involved in mitochondrial metabolism between the two types of species were identified. Overall, this study promotes the understanding of the roles of mitochondria in postharvest fruit quality maintenance and expands our recognition of plant mitochondrial proteomes.

## Results

### Isolation of high-purity mitochondria from citrus pulp

**Fig. 1:**
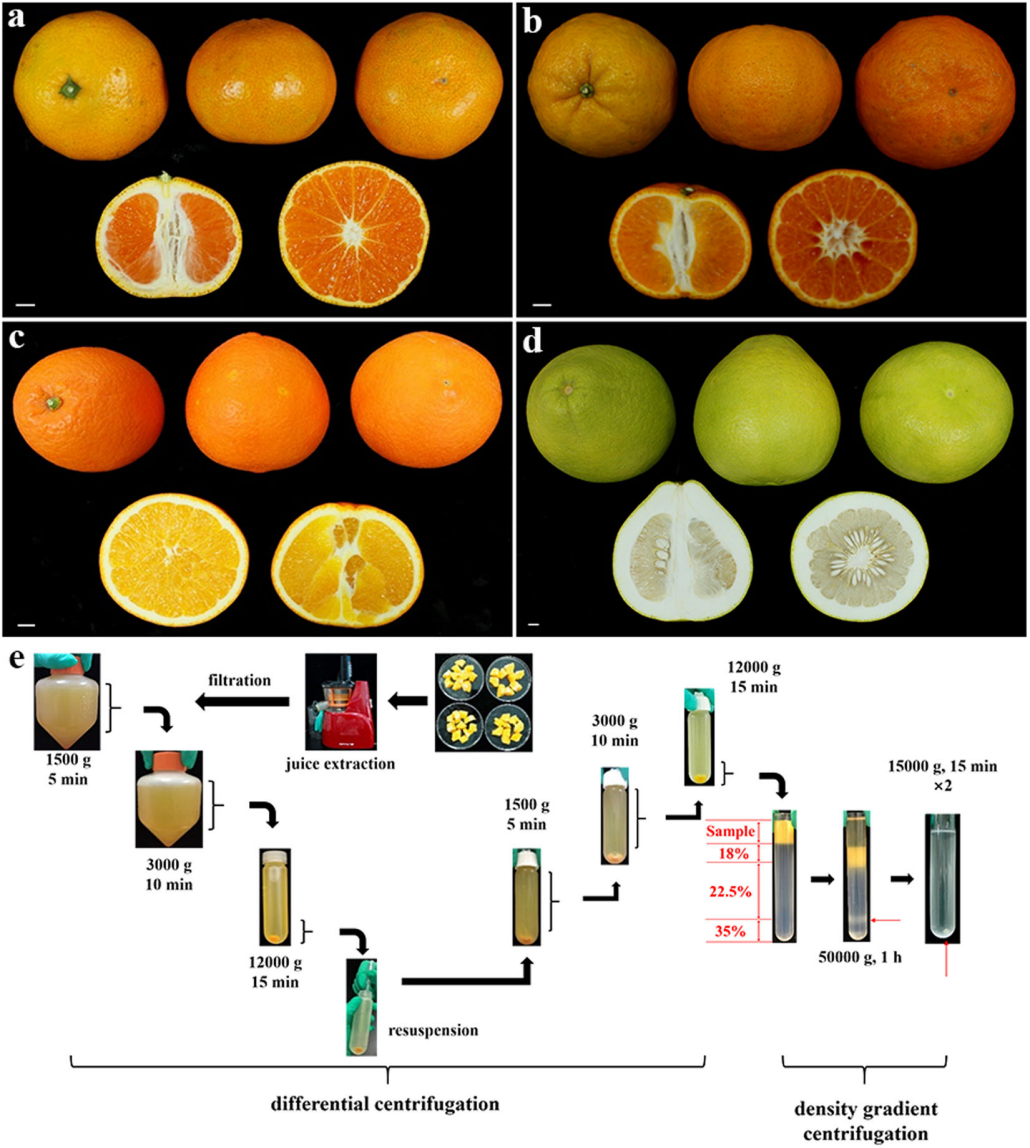
Workflow diagram for the isolation and purification of mitochondria from the pulp of different citrus species. **a–d** Anatomic structures of four citrus species used for mitochondrial preparation: (**a**) satsuma mandarin, (**b**) ponkan mandarin, (**c**) sweet orange, and (**d**) shatian pummelo. Bars = 10 mm. **e** Differential and Percoll density gradient centrifugation procedures for preparing highly pure mitochondria from the pulp of different citrus species. Tissue homogenization, formulation of extraction buffer, and centrifugation steps were optimized to obtain high-quality mitochondria

Isolation of pure mitochondria free from contamination by other organelles is crucial for subsequent subproteome analysis. In the present study, traditional differential and Percoll density gradient centrifugation were used to isolate and purify mitochondria from the pulp of four citrus species (Fig. 1). Considering the high levels of primary and secondary metabolites in citrus fruits, we improved tissue homogenization (Supplementary Fig. S1a–c) and extraction buffer and performed two rounds of differential centrifugation to reduce contamination from other organelle fractions as much as possible prior to Percoll gradient centrifugation. In particular, the buffering capacity, pH, and osmotic pressure of the extraction buffer were optimized to maintain the integrity of the mitochondrial membrane during homogenization. Additionally, a peristaltic pump was used to collect the supernatants and to reduce the loss of mitochondrial pellets during differential centrifugation (Supplementary Fig. S1d, e). Subsequently, the crude mitochondrial fraction was further purified by density gradient centrifugation, and the mitochondrial band was enriched at the 22.5–35% Percoll gradient interface (Fig. 1e). After recovery, the mitochondrial fraction was washed twice with washing buffer.

**Fig. 2:**
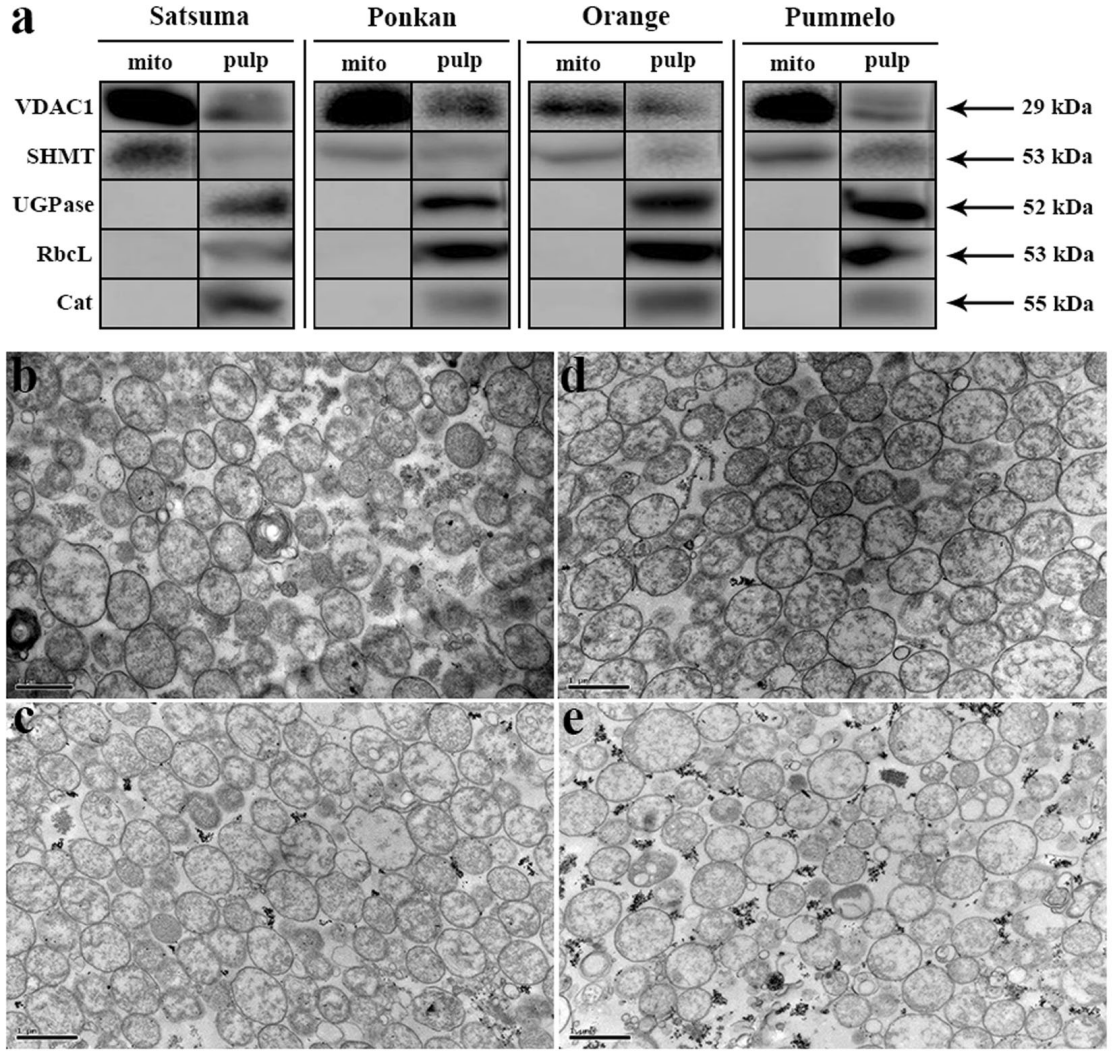
Purity evaluation of isolated mitochondria. **a** Western blot analysis of mitochondrial enriched fractions (mito) and total pulp protein extracts (pulp) of different citrus species. A total of 10 μg of mitochondrial proteins and pulp proteins were separated by 10% SDS-PAGE and blotted onto PVDF membranes. Blots were performed using different marker antibodies against VDAC1 (mitochondrial outer membrane), SHMT (mitochondrial matrix), UGPase (cytoplasm), RbcL (plastid), and Cat (peroxisome). Uncropped blot images are shown in Supplementary Fig. S6. **b–e** Transmission electron microscopy (TEM) images of isolated mitochondria from (**b**) satsuma mandarin, (**c**) ponkan mandarin, (**d**) sweet orange, and (**e**) shatian pummelo. Bars = 1 μm

The purity of the isolated mitochondria was assessed by western blot analysis using polyclonal antibodies against different marker proteins for different subcellular compartments, including mitochondria, cytoplasm, plastids, and peroxisomes (Fig. 2a). As expected, the mitochondrial marker proteins VDAC1 (mitochondrial outer membrane) and SHMT (mitochondrial matrix) were readily detected in the samples extracted from the isolated mitochondria; in contrast, the marker proteins predicted to be localized to the cytosol (UGPase), plastids (RbcL), and peroxisomes (Cat) could not be detected (Fig. 2a), indicating that there was no detectable cytosolic, plastidic, or peroxisomal contamination in the isolated mitochondria. However, all these marker proteins could be detected in the total pulp protein extracts of the corresponding species (positive control) (Fig. 2a). In addition, transmission electron microscopy (TEM), optical microscopy, and confocal microscopy were also utilized to evaluate the purity of the isolated mitochondria (Fig. 2b–e and Supplementary Fig. S2). As a result, the isolated mitochondria exhibited intact membranes and dense matrices without visible contamination by plastids or peroxisomes. These results suggested that the prepared mitochondria were of high purity and could be used for subsequent subproteomic analysis.

### Characterization of mitochondrial proteomes of citrus fruit

**Fig. 3:**
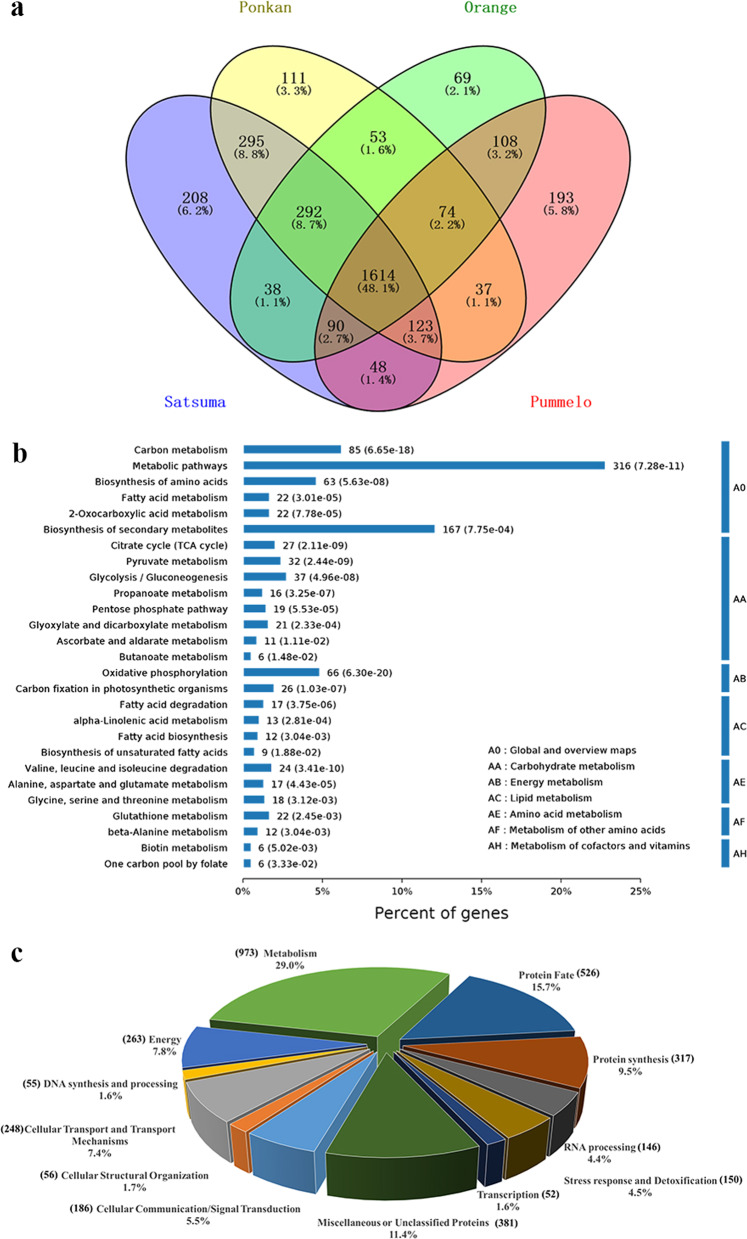
Characterization and functional classification of four citrus mitochondrial proteomes. **a** Venn diagram of the overlapping proteins identified from at least two biological replicates in each species. A total of 1614 proteins were commonly identified in four species. Detailed information on 3353 nonredundant proteins identified from the four species is given in Supplementary Table S2. **b** KEGG analysis of the 1614 overlapping proteins indicated in (**a**). Pathways related to energy metabolism, such as oxidative phosphorylation, TCA cycle, and pyruvate metabolism, were significantly enriched. **c** Functional distribution of the 3353 proteins identified in this study based on the functional classification of Heazlewood et al.^6^. These proteins can be divided into 12 major functional groups and mainly participate in metabolism (29.0%), protein fate (15.7%), protein synthesis (9.4%), energy (7.8%), and cellular transport and transport mechanisms (7.4%)

A label-free LC-MS/MS-based proteomic approach coupled with highly sensitive Q-Exactive MS was employed to characterize the mitochondrial proteome of citrus fruit. To maximize the outcome of protein identification, three citrus protein databases were integrated (see Materials and Methods). As a result, a total of 26,740 assigned peptides representing 3755 nonredundant proteins were identified (Supplementary Table S1). Among them, 2708, 2599, 2338, and 2287 proteins were identified in at least two biological replicates of *Citrus unshiu*, *Citrus reticulata*, *Citrus sinensis*, and *Citrus grandis*, respectively, resulting in a total of 3353 proteins for further analysis (Supplementary Tables S1 and S2). There were 1614 proteins commonly detected in all four species, accounting for 48.1% of all identified proteins, and these proteins were significantly enriched in carbon metabolism, TCA cycle, pyruvate metabolism, and oxidative phosphorylation, as indicated by Kyoto Encyclopedia of Genes and Genomes (KEGG) analysis (Fig. 3a, b).

For a better understanding of the metabolic pathways in mitochondria and exploration of novel mitochondrial proteins, all the identified proteins were assigned to 12 general functional categories according to the classification by Heazlewood et al.^6^. The major categories consisted of proteins involved in metabolism (29.0%), protein fate (15.7%), protein synthesis (9.4%), energy (7.8%), cellular transport and transport mechanisms (7.4%), and cellular communication/signal transduction (5.5%) (Fig. 3c and Supplementary Table S2). Similar distribution patterns of functional groups were observed for different species, indicating that mitochondrial metabolism is generally conserved (Table 1). It is worth noting that several proteins with ambiguous annotations or unknown functions were categorized as miscellaneous or unclassified proteins, and some of these had no definite orthologs in other plants, indicating that a number of mitochondrial functions in citrus/plants have not been discovered (Fig. 3c, Table 1, and Supplementary Table S2). Subdivision of the largest functional group, metabolism, further revealed that a number of proteins were mainly involved in the metabolism of amino acids (16.8%), lipids (16.3%), coenzymes/cofactors (7.2%), and nucleotides (5.9%) (Supplementary Fig. S3).

**Table 1:** Functional distribution of the proteins identified in each citrus mitochondrial proteome

Function	*Citrus unshiu*		*Citrus reticulata*		*Citrus sinensis*		*Citrus grandis*	
	No. of Proteins	Percentage	No. of Proteins	Percentage	No. of Proteins	Percentage	No. of Proteins	Percentage
Cellular communication/signal transduction	151	5.6	145	5.6	113	4.8	126	5.5
Cellular structural organization	46	1.7	43	1.6	34	1.4	38	1.6
Cellular transport and transport mechanisms	209	7.7	202	7.8	165	7.1	162	7.1
DNA synthesis and processing	47	1.7	44	1.7	46	2.0	39	1.7
Energy	215	7.9	225	8.7	220	9.4	215	9.4
Metabolism	769	28.4	702	27.0	649	27.8	636	27.8
Protein fate	450	16.6	418	16.1	348	14.9	318	13.9
Protein synthesis	279	10.3	277	10.7	256	11.0	251	11.0
RNA processing	92	3.4	91	3.5	104	4.4	109	4.8
Stress response and detoxification	123	4.5	122	4.7	115	4.9	101	4.4
Transcription	37	1.4	36	1.4	37	1.6	44	1.9
Miscellaneous or unclassified proteins	290	10.7	294	11.3	251	10.7	248	10.8
Total	2708		2599		2338		2287	

The proteins identified in each citrus mitochondrial proteome were divided into 12 major functional groups based on the functional classification of Heazlewood et al.^6^. The number and proportion of proteins in corresponding functional groups are listed

Several mitochondrial metabolic pathways associated with citrus fruit quality development and maintenance were identified. For example, all known enzymes of the TCA cycle have been identified in different species of citrus fruit (Supplementary Table S2). In addition, several enzymes, such as glutamate decarboxylase (GAD), γ-aminobutyric acid transaminase (GABA-T), and succinic semialdehyde dehydrogenase (SSADH), which are potentially involved in GABA shunt metabolism, were detected in different species as well (Supplementary Table S2), indicating that this pathway plays a certain regulatory role in citric acid metabolism. Considering the high level of AsA (vitamin C) in citrus fruit, we also identified proteins related to AsA metabolism, including those related to biosynthesis (L-galactono-1,4-lactone dehydrogenase) and the AsA-GSH cycle (ascorbate peroxidase (APX), monodehydroascorbate reductase (MDHAR), dehydroascorbate reductase (DHAR) (Supplementary Table S2). Additionally, three key enzymes responsible for biotin (vitamin H or B8) biosynthesis, including 7,8-diaminopelargonic acid (DAPA) synthase/adenosylmethionine-8-amino-7-oxononanoate aminotransferase (DAPAS or DAPAAT), dethiobiotin synthetase (DBS), and biotin synthase (BS), were found in this study (Supplementary Table S2). Several enzymes were also found to be involved in the biosynthesis and transport of folate (vitamin B9, Supplementary Table S2), a process that also takes place in mitochondria. To summarize, the proteomics analysis results revealed a number of proteins that participate in mitochondrial metabolism and are closely associated with citrus fruit quality.

Furthermore, six classical subcellular targeting prediction programs, including TargetP, Predotar, MitoProtII, iPSORT, WoLF PSORT, and MU-LOC, were used to determine whether the identified proteins were mitochondrially localized. Overall, more than 50% of all the identified proteins were predicted to be mitochondrial by at least one program in all four species, while over 35% of the proteins in each species were not predicted to be mitochondrial by any program, suggesting that they may be peripherally associated with mitochondria or may not be targeted to the mitochondria (Supplementary Fig. S4a and Supplementary Table S2). It is worth noting that when multiple prediction tools were integrated, even less than 10% of proteins for each species were predicted to be mitochondrially localized by all six programs, suggesting a poor overlapping prediction under the combination of different prediction tools (Supplementary Fig. S4a). The accuracy of the six programs was also assessed. As shown in Supplementary Fig. S4b, the predicted sensitivities of these programs ranged from 10% (WoLF PSORT) to 45% (MitoProtII), while iPSORT and MU-LOC showed a slightly lower prediction sensitivity (approximately 40%), and an intermediate prediction sensitivity (approximately 25%) was observed between TargetP and Predotar.

### Quantitative analysis of the mitochondrial proteomes in citrus fruit

**Fig. 4:**
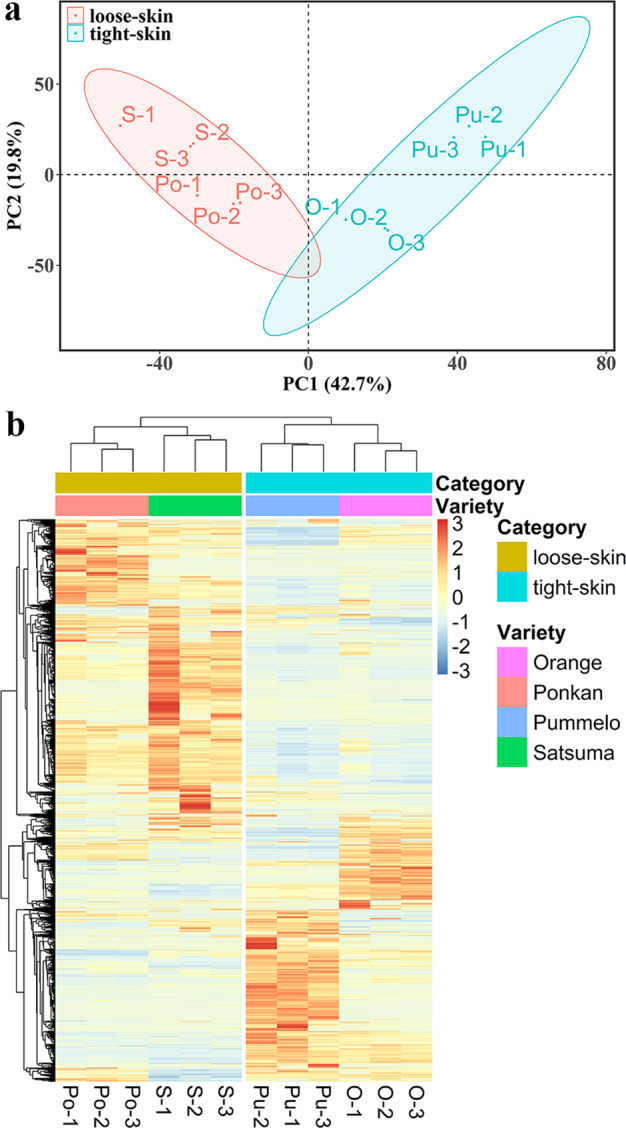
Principal component analysis and hierarchical clustering analysis of differentially abundant proteins among four citrus mitochondrial proteomes. **a** A total of 2716 DAPs were screened among four species using the fold change method (> 2 or < 0.5) and *P* value < 0.05 with Student’s *t*-test. Similar protein expression profiles were observed among three biological replicates for each species. **b** The protein expression patterns showed a relatively high degree of similarity between satsuma and ponkan, as well as between orange and pummelo. Detailed information on the 2716 DAPs between species is listed in Supplementary Table S3

Intensity-based absolute quantification (iBAQ)-based quantitative analysis was performed to further examine the differences among the four citrus mitochondrial proteomes. A total of 2716 DAPs were screened among the four species (Supplementary Table S3). Both principal component analysis (PCA) and hierarchical clustering analysis showed similar protein expression profiles for each species among the three biological replicates, indicating consistency in both the protein content and technical reproducibility of each experimental dataset (Fig. 4). Interestingly, the protein expression patterns showed a relatively higher degree of similarity between satsuma and ponkan and between orange and pummelo (Fig. 4b). In addition, we compared the mitochondrial proteomes of different combinations of species (Supplementary Table S4). There was a high degree of overlap between satsuma and ponkan (77.9%) and between orange and pummelo (68.9%), which is in agreement with the classification of loose-skin (satsuma and ponkan) and tight-skin (orange and pummelo) citrus fruits in commercial postharvest practices.

**Fig. 5:**
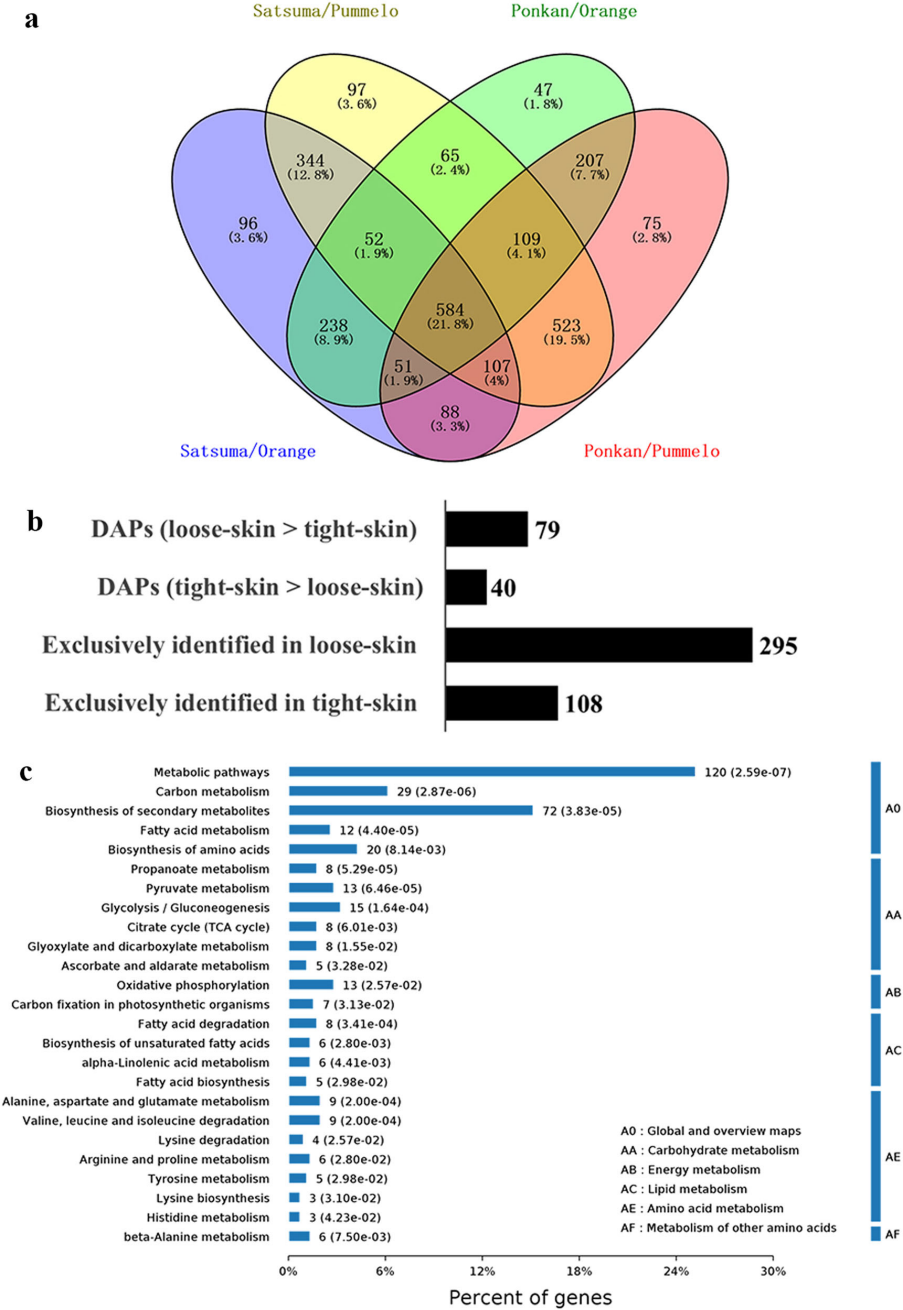
Comparison of mitochondrial proteomes between loose-skinned and tight-skinned citrus fruits. **a** Venn diagram of the overlapping DAPs in different combinations of species. There were 584 DAPs commonly detected in all four combinations. After the exclusion of the DAPs between species within the same category, a total of 522 proteins were screened as DAPs between loose-skin and tight-skin citrus fruits. **b** Number of DAPs between loose-skinned and tight-skinned citrus fruits. Detailed information on the 522 DAPs between loose-skinned and tight-skinned citrus fruits is listed in Supplementary Table S5. **c** KEGG analysis of DAPs between loose-skinned and tight-skinned citrus fruits. Pathways associated with fatty acid and amino acid metabolism were significantly enriched

**Fig. 6:**
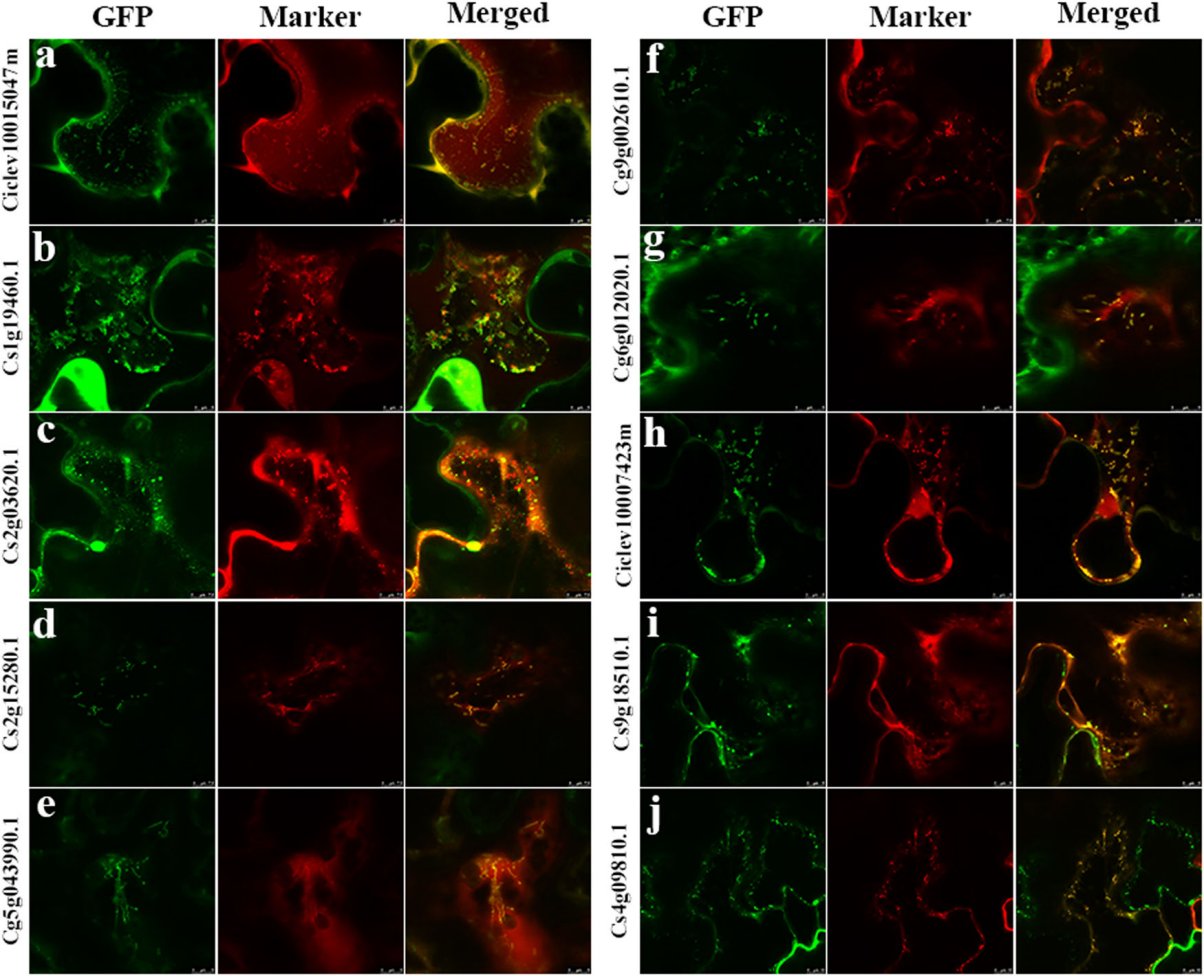
Subcellular localization study of ten selected proteins identified in this study. **a**–**j** Ten proteins identified in this study (Supplementary Table S6) were fused in frame with the GFP fluorescence tag and then transiently coexpressed with the mitochondrial marker in tobacco leaves. The left lane shows the GFP fluorescence signal, the center lane shows the mCherry fluorescence of the mitochondrial marker, and the right lane shows an overlay between the two types of fluorescence. Among them, Cs1g19460.1 (**b**) and Cs2g03620.1 (**c**) were likely to be partially mitochondria-localized or peripherally associated with mitochondria

We then further compared the mitochondrial proteomes between loose-skinned and tight-skinned citrus fruits. In total, 1560, 1881, 1353, and 1744 DAPs were identified between satsuma and orange, satsuma and pummelo, ponkan and orange, and ponkan and pummelo, respectively (Supplementary Table S3). Among the 584 DAPs commonly detected in all four combinations, a total of 522 proteins were recognized as the DAPs between loose-skin and tight-skin citrus fruits after excluding the DAPs between the species of the same type (i.e., satsuma and ponkan; orange and pummelo) (Fig. 5a and Supplementary Table S5). Compared with those in tight-skin citrus fruit, 79 and 40 DAPs exhibited significantly higher and lower abundances in loose-skin citrus fruit, respectively (Fig. 5b and Supplementary Table S5). In addition, 295 and 108 unique DAPs were exclusively identified in loose-skin and tight-skin citrus fruit, respectively (Fig. 5b and Supplementary Table S5). Notably, KEGG analysis showed that the majority of the pathways enriched in the 522 DAPs were associated with fatty acid and amino acid metabolism, implying significantly differential metabolic pathways between loose-skin and tight-skin citrus fruits (Fig. 5c). Several DAPs related to fatty acid metabolism (Ciclev10015047m, Cs1g19460.1, Cs2g03620.1) and amino acid metabolism (Cs2g15280.1, Cg5g043990.1, Cg9g002610.1) were confirmed to be localized to mitochondria in tobacco plants by transient expression of fluorescent fusion proteins, and a similar mitochondrial localization was also observed for proteins related to GABA shunt metabolism (Cg6g012020.1), and biosynthesis of biotin (Ciclev10007423m) and folate (Cs9g18510.1), verifying the involvement of mitochondria in primary and secondary metabolism (Fig. 6 and Supplementary Table S6).

## Discussion

This study establishes an optimized protocol for obtaining highly pure mitochondria from citrus pulp, followed by a large-scale characterization of the mitochondrial proteomes of four major citrus species. A further quantitative comparison of the mitochondrial proteomes of loose-skinned and tight-skinned fruits was then carried out, providing new insights into the differences in metabolic pathways related to fruit quality maintenance at the subcellular level.

### Isolation of highly pure mitochondria from the pulp of various citrus fruits

Research on the physiological and biochemical functions of mitochondria often requires the isolation of intact and active mitochondria. It has been reported that mitochondria can be extracted from various plant materials, such as fleshy roots or tubers, etiolated seedlings, green tissues (leaves), and cell suspension cultures^11,12^. However, typical isolation protocols involve some time-consuming centrifugation steps and substantial amounts of starting materials. To overcome these limitations, very recently, a rapid isolation technique involving affinity purification of tagged mitochondria from *Arabidopsis* was developed, by which highly pure and intact mitochondria can be obtained^14–16^. However, it remains very difficult to isolate mitochondria of high purity and intactness from fleshy fruits, and particularly from the pulp, due to the high levels of primary and secondary metabolites in these tissues. Additionally, the difficulty in obtaining stably transformed citrus fruit also hinders the application of affinity purification to the isolation of mitochondria. Therefore, it is necessary to optimize the existing methods for obtaining highly pure and intact mitochondria from citrus fruit.

Centrifugation-based methods have been widely used to isolate mitochondria from various fruit species. However, we failed to isolate mitochondria of high purity and intactness from citrus fruit using published protocols optimized for other species (data not shown). For fleshy fruit with a high water content, the preparation of highly pure mitochondria is rather challenging because of the relatively low abundance of mitochondria as well as high levels of organic acids, sugars, phenols, and pigments in fruit tissues, which may seriously interfere with mitochondrial isolation and purification^13^. Hence, a new protocol was developed for the effective isolation of high-quality mitochondria from citrus pulp, which involves improvements in four steps: (1) A low-speed (50 rpm) juice extractor (JYZ-V5, Joyoung, China) was used to reduce the number of broken mitochondria and avoid frothing during homogenization. The juice and pomace were automatically separated during homogenization using this machine, which was conducive to the following filtration (Supplementary Fig. S1a–c). In the preliminary experiment, we found that homogenization with a Waring blender was too violent even at low speed, and a large amount of bubbles were produced, which significantly slowed the speed of subsequent filtration. (2) Under acidic conditions, proteases are active, and mitochondria are more susceptible to damage^37^. Considering the high level of citric acid in the juice sac cells of citrus pulp, the 3-(N-morpholino) propanesulfonic acid (MOPS) content was elevated to 200 mM and 50 mM in the extraction buffer and washing buffer, respectively. To reduce medium acidification caused by the release of vacuolar content, the pH of the extraction buffer was adjusted to 7.8–8.0 before homogenization, ensuring that the overall pH stayed above 7.0 during the whole preparation process. In fact, adjusting the pH after homogenization may be too late, as mitochondria are likely damaged during the process of isolation. (3) To prevent mitochondrial aggregation during the preparation process, an isolation medium composed of MOPS and sorbitol was used^40^. Importantly, the pH of the extraction and washing buffers was adjusted with Tris rather than with KOH or NaOH. (4) After differential centrifugation, a peristaltic pump was utilized not only to avoid pellet contaminants when collecting the supernatant, but also to reduce the loss of crude mitochondria when decanting the supernatant (Supplementary Fig. S1d, e). Through these improvements, a universal protocol applicable to the isolation of mitochondria from the pulp of different citrus fruits was established.

Currently, although various methods, such as density gradient centrifugation and FFE, can be employed to further purify mitochondria, contamination from other compartments seems to be unavoidable, since proteins with different subcellular localizations may be copurified during the isolation process^3,6,7,41,42^. Thus, it is crucial to assess the purity of the prepared mitochondria prior to subsequent analysis. Various techniques have been used to determine the degree of contamination from various subcellular compartments, such as microscopy observation^43^, assays of marker enzyme activity^6^, and western blot analysis of target proteins^42^. We combined western blot analysis with microscopy observations to determine the purity of the isolated mitochondria (Fig. 2 and Supplementary Fig. S2). No detectable contamination was observed from other subcompartments, including cytosol, plastid, and peroxisome contamination, and the mitochondrial membrane structure remained intact, indicating that the isolated mitochondria were of high purity and retained their activity, and thus could be used for the subsequent proteomic analysis.

Due to their high throughput and low cost, bioinformatic approaches provide an alternative approach to exploring mitochondrial proteins on a large scale^7^ and thus are often used to evaluate the purity of isolated mitochondria. Because each program may employ different algorithms for subcellular prediction, it is plausible that these programs (MitoProt II, TargetP, iPSORT, Predotar, WoLF PSORT, MU-LOC) produce relatively large and nonoverlapping sets of results with variable prediction sensitivities (Supplementary Fig. S4). Similar concerns have also been raised from previous reports in *Arabidopsis*, rice, and potato, indicating that pure bioinformatic approaches may have limited efficacy in the identification of mitochondrial proteins^7^. First, most programs perform subcellular prediction based on the recognition of the N-terminal cleavable peptide signal, which is known as a presequence that serves as a sorting signal to direct the protein to mitochondria^44^. However, for mitochondrial precursor proteins lacking N-terminal targeting presequences, such as twin cysteine proteins, β-barrel proteins, transporter proteins, and mitochondrially encoded proteins^42,44^, the prediction may result in low accuracy and sensitivity. In addition, for dual/multiple-targeted proteins that localize to more than two locations in plants^45^, prediction is much more complicated, leading to rather limited authenticity. Finally, as most of these computational tools were developed decades ago, the algorithms should be updated and optimized based on more comprehensive genomics and proteomics data, or new tools should be developed.

### Comparative analysis of mitochondrial proteomes among different citrus fruits

Proteomic analysis of the isolated mitochondria identified more than 2000 proteins for each citrus species (Supplementary Table S1), which is consistent with the estimated number of mitochondrial proteins in plants^2^. However, proteins of low abundance are likely to be detected as a result of the high sensitivity of the chosen LC-MS/MS identification technique. These proteins may represent previously unconfirmed mitochondrial proteins or potential contaminants from other organelles (Supplementary Table S2). In addition, several identified proteins had no clear functional categorization or were not homologous to any other proteins of known function (Table 1, Fig. 3c, and Supplementary Table S2). To obtain comprehensive and in-depth insights into potential novel functions and signaling pathways involving mitochondrial proteins, these experimentally identified proteins were all included for further analysis.

In all eukaryotic organisms, the fundamental function of mitochondria is to provide energy via the TCA cycle and oxidative phosphorylation^15^. A shortage of energy can affect membrane integrity and ROS production, leading to pericarp browning and ultimately affecting fruit quality. Therefore, mitochondrial energy metabolism plays an essential role in slowing the quality deterioration and senescence process of harvested fruits^46^. Additionally, organic acids are not only substrates produced during respiration, but also important precursors for primary metabolites (amino acids and fatty acids) and secondary metabolites, which contribute greatly to fruit flavor and quality^35^. Thus, it is not surprising that proteins involved in energy-related metabolic pathways, such as oxidative phosphorylation, TCA cycle, and pyruvate metabolism, were significantly enriched in all species (Fig. 3a, b and Supplementary Table S2).

For commercial postharvest practices, according to the significant differences in the tightness of flesh-rind anatomic structure, citrus fruits can be classified into loose-skin (easy to peel) citrus fruit such as satsuma mandarin (*C. unshiu*) and ponkan mandarin (*C. reticulata*), and tight-skin (hard to peel) citrus fruit such as sweet orange (*C. sinensis*) and pummelo (*C. grandis*)^39^. These two categories of citrus fruit differ greatly in storage performance and can be used as models for studies of fruit ripening and senescence^39^. Overall, tight-skin citrus fruit has a longer storage life than loose-skin fruit. To further elucidate the differences among different citrus species at the subcellular level, a quantitative analysis of the four mitochondrial proteomes was performed. The protein expression patterns were highly conserved between species within the same category (e.g., satsuma and ponkan; sweet orange and pummelo) (Fig. 4 and Supplementary Table S4). Therefore, the expression profile of mitochondrial proteins may be a critical factor that can be used to distinguish loose-skinned and tight-skinned citrus fruit. KEGG analysis further revealed that the DAPs were mainly involved in fatty acid and amino acid metabolism, which may explain the difference in fruit quality maintenance and storage life between the two categories (Fig. 5c).

**Fig. 7:**
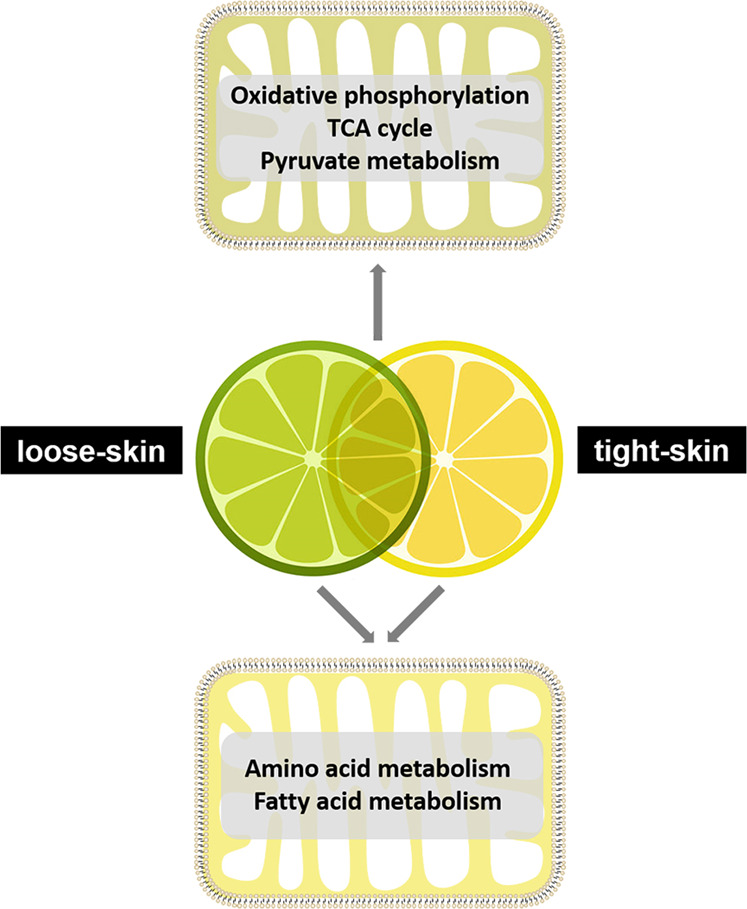
Major similarities and differences in mitochondrial metabolism between loose-skinned and tight-skinned citrus fruits. Proteins identified in all species were mainly involved in oxidative phosphorylation, the TCA cycle and pyruvate metabolism, indicating that mitochondrial energy metabolism is conserved among different citrus fruits. The DAPs between loose-skinned and tight-skinned citrus fruits were mainly associated with fatty acid and amino acid metabolism, implying differences in mitochondrial metabolism between the two types of citrus fruit

Fatty acids and amino acids play key biological roles in fruit and are major precursors of aroma volatiles in several fruits^47^, which may be related to off-flavor production during citrus fruit storage^48^. In plants, although fatty acid biosynthesis and degradation (β-oxidation) mainly occur in plastids (chloroplasts) and peroxisomes, respectively, there has been increasing evidence showing the involvement of mitochondria in fatty acid metabolism^7,42,49–51^. In the present study, two DAPs involved in fatty acid β-oxidation were confirmed to be fully or partially mitochondrially localized by in vivo subcellular targeting analysis (Fig. 6a, b), while their *Arabidopsis* orthologs At4g04320 (malonyl-CoA decarboxylase) and At1g04290 (a thioesterase superfamily protein) were both identified in the leaf peroxisome proteome and verified by transient fluorescence assay in tobacco plants (for At4g04320)^52^. As the key enzyme in fatty acid synthesis, β-ketoacyl-[acyl carrier protein] synthase I (KASI) participates in the elongation of the carbon chain and plays an important role in chloroplast division^53^, while its citrus ortholog identified in this study was also confirmed to be partially localized to mitochondria (Fig. 6c). The site of amino acid catabolism in plants is relatively complicated and involves multiple subcellular compartments, including the mitochondria, cytosol, plastids, and peroxisomes^54^. Three DAPs involved in the degradation of aspartate (Fig. 6d) and branched-chain amino acids (Fig. 6e, f) were all confirmed to be mitochondrially localized by green fluorescent protein (GFP) studies, which is consistent with the localization of their *Arabidopsis* orthologs (Supplementary Table S2)^54^. In addition, several proteins potentially related to fruit quality and participating in GABA shunt metabolism (Fig. 6g), and biosynthesis of biotin (Fig. 6h) and folate (Fig. 6i) all showed mitochondrial localization, as indicated by the in vivo subcellular targeting analysis (Supplementary Table S6). Notably, a protein without clear functional annotation or *Arabidopsis* ortholog was also verified to be localized to mitochondria (Fig. 6j), suggesting that it is a novel mitochondrial protein. Taken together, our experimental results highlight the major similarities and differences in mitochondrial metabolism between loose-skinned and tight-skinned citrus fruits (Fig. 7) and expand the plant mitochondrial proteome.

### Comparison of mitochondrial proteomes among plant species

To gain new insights into plant mitochondrial metabolism, the mitochondrial proteomes of *Arabidopsis*^7,55^, rice^3^, potato^42^, maize^56^, and citrus (this study) were categorized into 12 general functional groups according to the classification by Heazlewood et al.^6^ (Supplementary Fig. S5). Overall, the functional categories of metabolism, energy, protein fate, and protein synthesis accounted for relatively large proportions of all mitochondrial proteomes, suggesting that these biological processes are conserved and essential in mitochondria. The percentages of proteins related to protein fate, cellular transport and transport mechanisms, and cellular communication/signal transduction in the citrus mitochondrial proteome were higher than those in the other four plants. The percentage of proteins related to energy in citrus was significantly lower than that in maize and rice, possibly because mitochondria from mature fruit tissues are less active in energy production, whereas maize seed embryos and rice shoots are highenergy-consuming organs, thus reflecting the tissue-specific difference in the energy metabolism of different plant species (Supplementary Fig. S5a). Notably, a large number of proteins with ambiguous annotations or unknown functions were identified in each mitochondrial proteome; functional characterization of these proteins will improve our understanding of mitochondrial functions in plants (Supplementary Fig. S5a). KEGG analysis further revealed that the pathways of the TCA cycle, oxidative phosphorylation, carbon metabolism, fatty acid and amino acid metabolism, and biosynthesis of secondary metabolites (folate and biotin) were enriched in all species, confirming that mitochondria participate widely in primary and secondary metabolism (Supplementary Fig. S5b).

## Conclusion

By optimizing the buffer formulation, tissue homogenization, and centrifugation steps, a universal protocol was developed to isolate and purify mitochondria from the recalcitrant pulp tissue of four citrus species, allowing the high-throughput characterization of their mitochondrial proteomes and identification of conserved and unique metabolic pathways that may contribute to the differential quality attributes and postharvest storage performance between species. A comparison of the mitochondrial proteomes among different plant species further highlighted the involvement of mitochondria in primary and secondary metabolism. Collectively, our study provides a methodological basis for characterizing the role of mitochondria in fruit quality and exploring novel mitochondrial metabolic pathways in plants.

## Materials and methods

### Plant materials

Commercial mature satsuma mandarin (*C. unshiu* cv. Guoqing No. 1), ponkan mandarin (*C. reticulata* cv. Egan No.1), and sweet orange (*C. sinensis* cv. Newhall) fruits were collected in Yichang, Hubei Province, China, and shatian pummelo (*C. grandis* cv. Guiyou No. 1) in Guilin, Guangxi Province, China, in 2017 in the harvest season (Fig. 1a–d). Fruits with uniform color, shape and size, and without physical injuries or diseases were randomly selected and stored at 4 °C until use.

### Isolation of mitochondria from citrus fruits

Mitochondria were isolated from citrus pulp by the method of Millar et al.^57^ and Wu et al.^29^ with some modifications, by utilizing differential centrifugation in conjunction with discontinuous Percoll gradients for density gradient centrifugation (Fig. 1e). All procedures were performed at 4 °C. Prior to isolation of mitochondria, the peels and seeds of fruits were removed, and approximately 90 g of pulp was cut into small pieces and immersed in 250 mL extraction buffer (0.4 M sorbitol, 0.2 M MOPS-Tris [pH 7.8], 7.5 mM EDTA, 1.5% [w/v] PVP-40, 0.1% [w/v] bovine serum albumin, and 2 mM DTT) for 5 min (Katz et al., 2007). Peeled pulps were homogenized in extraction buffer using a juice extractor (JYZ-V5, Joyoung, China; Supplementary Fig. S1a–c) three times for 20 s each with 10 s intervals. The homogenate was filtered through six layers of gauze, and the pooled filtrate was subsequently centrifuged for 5 min at 1500*g* to remove the starch, nuclei, and cell debris. The supernatant was collected and then centrifuged at 3000*g* for 10 min to remove the remaining denser particles. The supernatant was recovered and centrifuged at 12,000*g* for 15 min. The resulting high-speed supernatant was discarded, and the organelle pellet was resuspended in washing buffer (0.33 M sorbitol, 50 mM MOPS-Tris [pH 7.5]) with the aid of a 1 mL pipette. These centrifugation steps were repeated once to further reduce contamination from other organelles, and the resulting pellet was resuspended in a small volume of washing buffer. The crude mitochondrial fraction was layered onto a three-step Percoll gradient of 18%, 22.5%, and 35% [v/v] (2/6/3 mL) in washing buffer; the Percoll concentrations had been optimized for the isolation of mitochondria from citrus fruit in a series of preliminary experiments. After ultracentrifugation at 50,000*g*_avg_ for 60 min in a swing-out rotor (CP80NX, Hitachi, Japan), the mitochondrial band was enriched at the 22.5–35% Percoll gradient interface and recovered using a 10 mL syringe fitted with a 0.80 × 120 mm needle. Then, the mitochondrial band was diluted 10-fold with washing buffer, followed by centrifugation for 15 min at 15,000*g* to remove the Percoll. The washing step was repeated once, and the purified mitochondria were finally resuspended in a small volume of washing buffer.

### Mitochondrial protein extraction

Mitochondrial proteins were extracted by the method of Huang et al.^11^ with minor modifications. Purified mitochondria were precipitated by adding a 5× volume of cold acetone, followed by incubation at −20 °C overnight. The precipitated sample was spun at 17,000*g* for 15 min at 4 °C. The resulting supernatant was discarded, and the pellets were dried at room temperature (RT) for 15 min. The pellets containing mitochondrial proteins were then resuspended in an appropriate volume of STD buffer (4% [w/v] SDS, 100 mM Tris-HCl [pH 7.6], 1 mM DTT). The resuspended samples were spun at 17,000*g* for 10 min at 4 °C to remove any insoluble material. The supernatant was transferred to a new tube for western blot or LC-MS/MS analysis. The protein concentration was determined using a bicinchoninic acid protein assay kit (Beyotime).

### Purity assessment of the prepared mitochondria

The purity of isolated mitochondria was assessed by western blot analysis and microscopy observation. For western blot analysis, polyclonal antibodies from Agrisera were used at the appropriate dilutions, as follows: UDP-glucose pyrophosphorylase (UGPase; cytoplasm marker, 51.6 kDa, 1:1000), Rubisco large subunit (RbcL; plastid marker, 52.7 kDa, 1:5000), catalase (Cat; peroxisomal marker, 55 kDa, 1:1000), voltage-dependent anion-selective channel protein 1 (VDAC1; mitochondrial outer membrane, 29 kDa, 1:5000) and serine hydroxymethyltransferase (SHMT; mitochondrial matrix, 53 kDa, 1:5000). A total of 10 μg of fruit pulp or mitochondrial proteins were separated by 10% SDS-PAGE and blotted onto PVDF membranes using semidry transfer (Bio-Rad) according to the manufacturer’s instructions. Blots were blocked with 5% (w/v) skimmed milk powder in TBST buffer (20 mM Tris-HCl [pH 7.5], 150 mM NaCl, 0.05% [v/v] Tween-20) for 1 h at RT with agitation and subsequently incubated for 1 h at RT with agitation in the primary antibodies diluted as indicated above in TBST buffer containing 2% (w/v) skimmed milk powder. The antibody solution was decanted, and the blot was washed six times for 10 min in TBST at RT with agitation. Antibody-bound proteins were detected using the Clarity Western ECL Substrate Kit (Bio-Rad) after incubation in secondary antibody (anti-rabbit IgG horseradish peroxidase conjugated, Abbkine) diluted to 1:20,000 in TBST buffer with 2% (w/v) skimmed milk powder for 1 h at RT with agitation and washed as above. At least two independent replicates were performed for each antibody. For optical microscopy detection, the isolated mitochondrial pellet was stained with 0.02% [w/v] Janus Green B (Sigma) in Ringer’s solution (0.85% [w/v] NaCl, 0.25% [w/v] KCl, 0.03% [w/v] CaCl_2_). After incubation for 20 min at RT, mitochondrial morphology was observed with a microscope (DP70, Olympus). For confocal microscopy examination, the purified mitochondria suspended in washing buffer were stained with 500 nM MitoTracker Red CMXRos (M7512, Invitrogen) by the method of Pandey et al.^58^ with minor modifications. After incubation for 15 min at RT under dark conditions, images were captured by utilizing laser scanning confocal microscopy (TCS SP8, Leica) with excitation (552 nm) and emission (560–630 nm) wavelengths appropriate for MitoTracker Red dye. Samples for TEM analysis were sequentially fixed, dehydrated, embedded, stained, and observed with a TEM (H-7650, HITACHI) at an accelerating voltage of 70 kV.

### Sample preparation and LC-MS/MS

Protein (100 μg for each sample) digestion was performed based on the filter-aided sample preparation (FASP) method as described previously^59^, and the peptide content was then estimated by UV light spectral density at 280 nm using an extinction coefficient of 1.1 of 0.1% (g/L) solution that was calculated on the basis of the frequency of tryptophan and tyrosine in vertebrate proteins. NanoLC-MS/MS analysis was performed on an Easy-nLC system equipped with a Q-Exactive mass spectrometer (Thermo Fisher Scientific) as described by Sun et al.^60^.

### Database searching and protein identification

The maxQuant software package (version 1.5.3.17) was used to analyze the acquired tandem MS spectra for peptide identification and protein quantification. The original MS data were searched against the nonredundant protein databases of *C. sinensis* (http://citrus.hzau.edu.cn), *Citrus clementina* (https://phytozome.jgi.doe.gov/pz/portal.htmL), and *C. grandis* (http://citrus.hzau.edu.cn). The main parameters used for protein identification and quantitative analysis are listed in Supplementary Table S7. Raw data for peptide and protein identification are presented in Supplementary Table S8. Only the proteins identified in at least two biological replicates were retained for further quantification. DAPs between the two groups were screened using the fold change method (>2 or <0.5) and *P* value < 0.05 with Student’s *t*-test. Protein annotation information was obtained from the databases mentioned above.

### Bioinformatic analyses

Venn diagrams, PCA, hierarchical clustering analysis, and KEGG pathway analyses were performed using the Venny (https://bioinfogp.cnb.csic.es/tools/venny/index.html), factoextra, and pheatmap packages in R and the online multiomics data analysis toolkit OmicsBean (http://www.omicsbean.cn/), respectively. Predictions of subcellular localization for the identified proteins were performed for the full-length protein sequences in six programs: TargetP version 1.1 (http://www.cbs.dtu.dk/services/TargetP-1.1/index.php; Emanuelsson et al.^61^), Predotar version 1.04 (http://urgi.versailles.inra.fr/predotar/; Small et al.^62^), MitoProtII (http://ihg.gsf.de/ihg/mitoprot.html; Claros and Vincens^63^), iPSORT (http://ipsort.hgc.jp/; Bannai et al.^64^), WoLF PSORT (https://wolfpsort.hgc.jp/; Horton et al.^65^), and MU-LOC (http://mu-loc.org/; Zhang et al.^66^). The general function of each protein was assigned to a general functional category according to Heazlewood et al.^6^.

### Subcellular localization analysis

The coding sequences (CDSs) of the selected proteins (with the stop codon omitted) were cloned into the mt-gb^67^ vector with a GFP tag at SpeI and BamHI restriction sites under the control of the cauliflower mosaic virus 35S (CaMV 35S) promoter. For the colocalization assay, the GFP fusion construct was mixed with a mitochondrial marker (COX4ts fused to mCherry) and cotransformed into tobacco (*Nicotiana benthamiana*) leaves by *Agrobacterium tumefaciens* infiltration based on a previous description^68^. For imaging, GFP and mCherry signals were captured using laser scanning confocal microscopy (Leica TCS-SP8, Germany) in multitrack line switch mode. The primers used for gene cloning are listed in Supplementary Table S9.

## Supplementary information

Table S8.

Supplementary information (rewritten).

Figure S1.

Figure S2.

Figure S3.

Figure S4.

Figure S5.

Figure S6.

Table S2.

Table S3.

Table S5.
