# Supplementary material for: Isolation and comparative proteomic analysis of mitochondria from the pulp of ripening citrus fruit
Source: Hortic Res. 2021 Feb 1;8:31. doi: 10.1038/s41438-021-00470-w (PMC7848011; doi:10.1038/s41438-021-00470-w)
Supplement: Supplementary file 2 — Supplementary information (rewritten). [file 41438_2021_470_MOESM2_ESM.docx]

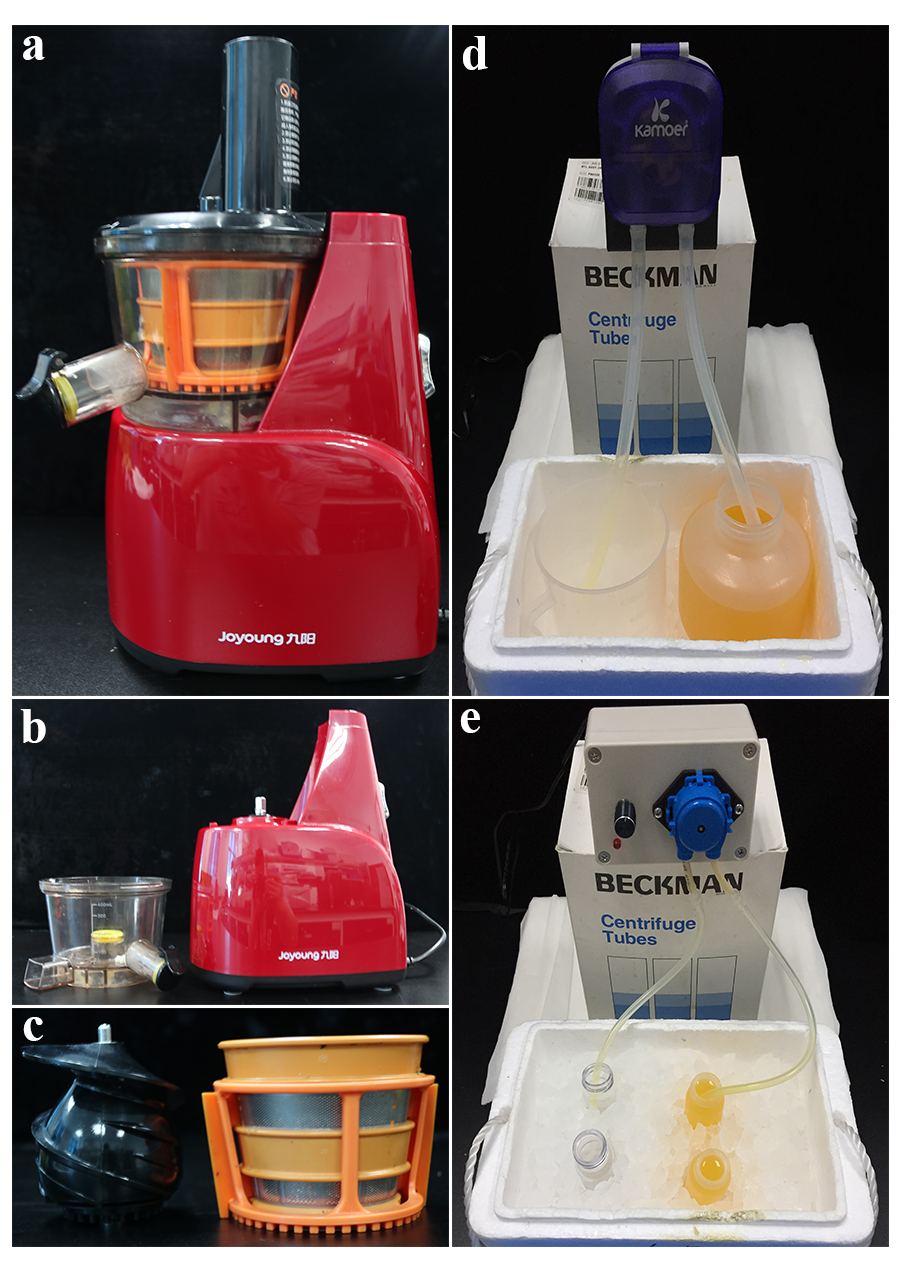


**Figure S1.** **Application of low-speed juice extractor and peristaltic pump in mitochondrial preparation.** **(a)–(c)** Detailed structure of juice extractor used in this study. Due to the low speed (50 rpm) during tissue homogenization, this juice extractor effectively avoided frothing. Additionally, the juice and pomace were automatically separated using this machine, thus facilitating the following filtration. **(d)–(e)** Application of peristaltic pump during differential centrifugation. (d) When collecting the supernatant during differential centrifugation, the peristaltic pump was utilized to avoid pellet contaminants; (e) when decanting the supernatant, it was used to reduce the loss of crude mitochondrial pellet.


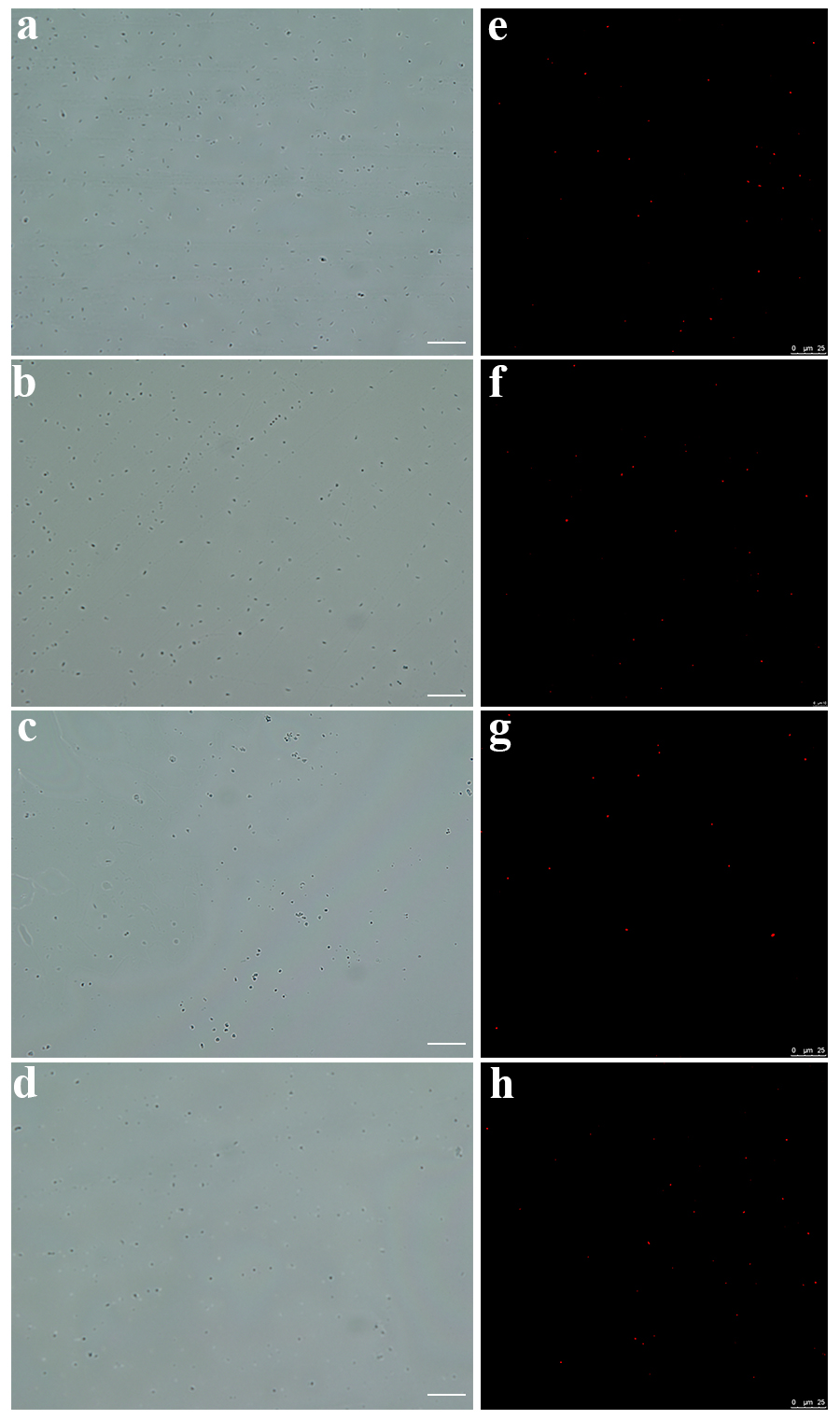


**Figure S2.** **Janus Green B staining and confocal imaging of mitochondria isolated from different citrus fruits.** (a) and (e), Satsuma mandarin; (b) and (f), ponkan mandarin; (c) and (g), sweet orange; (d) and (h), Shatian pummelo. The purified mitochondria were stained with Janus Green B (a–d, Bars = 10 μm) and MitoTracker Red (e–h) for indicated time, then the images were captured by optical microscopy and confocal microscopy, respectively.


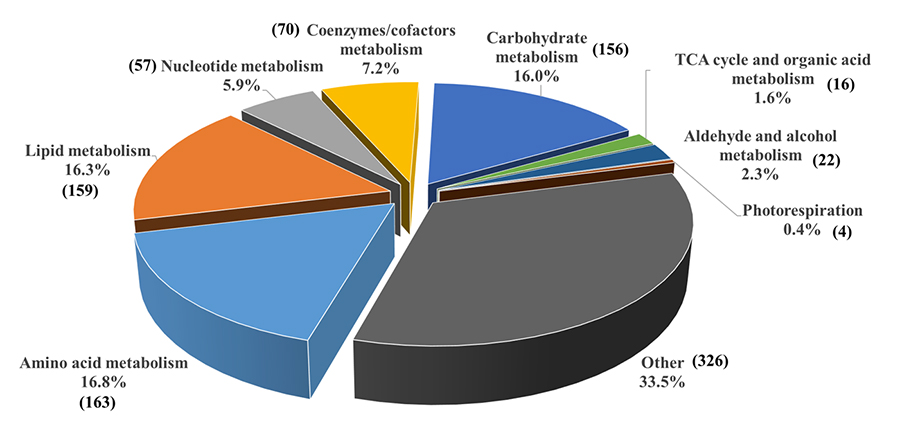


**Figure S3.** **Functional sub-distribution of proteins associated with metabolism.** A total of 973 proteins associated with the functional group of metabolism (Supplemental Table S2) indicated in Fig. 3c were further divided into several functional categories according to their annotations.


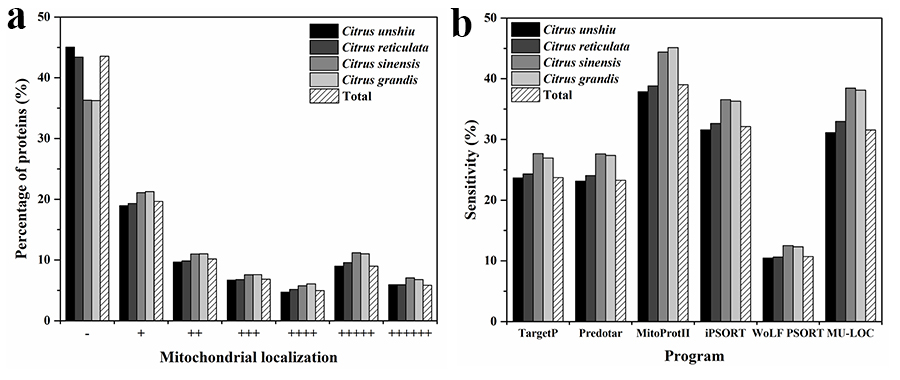


**Figure S4.** **Mitochondrial localization prediction by different programs.** **(a)** Mitochondrial localization prediction was performed by TargetP, Predotar, MitoProtII, iPSORT, WoLF PSORT and MU-LOC. The plus signs indicate prediction to be mitochondrial-localized by one to six programs, and the minus sign indicates prediction by none. The percentage of proteins predicted to be mitochondrially localized or not were shown in corresponding dataset. Combination details of the predictions are given in Supplemental Table S2. **(b)** Performance comparison of the six subcellular programs applied in different citrus fruits. Sensitivity is the percentage of positive predictions in corresponding species produced by each program.


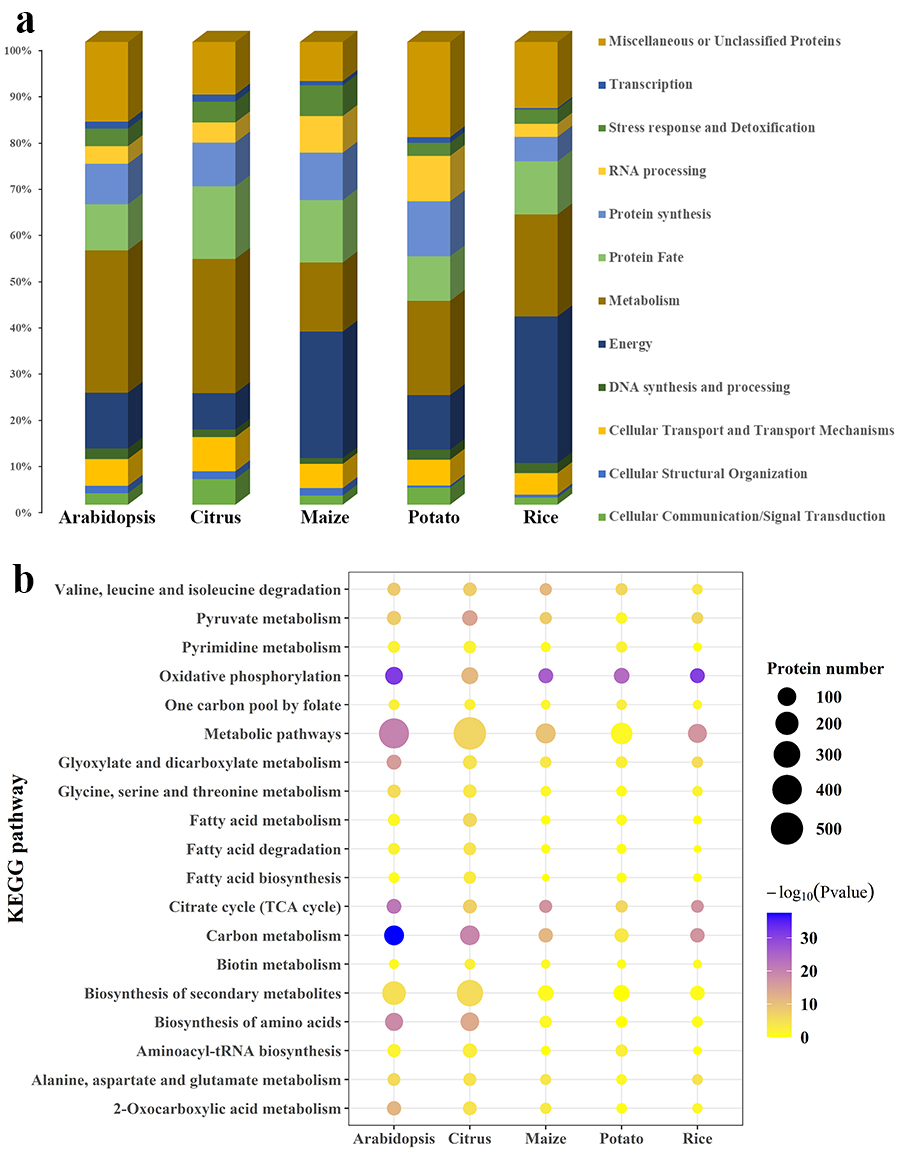


**Figure S5.** **Comparison of mitochondrial proteomes among plant species. (a)** Distribution of the five mitochondrial proteomes across functional categories according to Heazlewood et al.^6^. The corresponding functional classification of Arabidopsis (1736), rice (322), potato (1060) and maize (556) mitochondrial proteomes were generated according to Rao et al.^7^, Senkler et al.^55^ and Wang et al.^56^. **(b)** KEGG analysis of the five mitochondrial proteomes. The circle size indicates the number of proteins enriched in corresponding metabolic pathway, and the color of circle indicates the significance level of the enriched pathway.


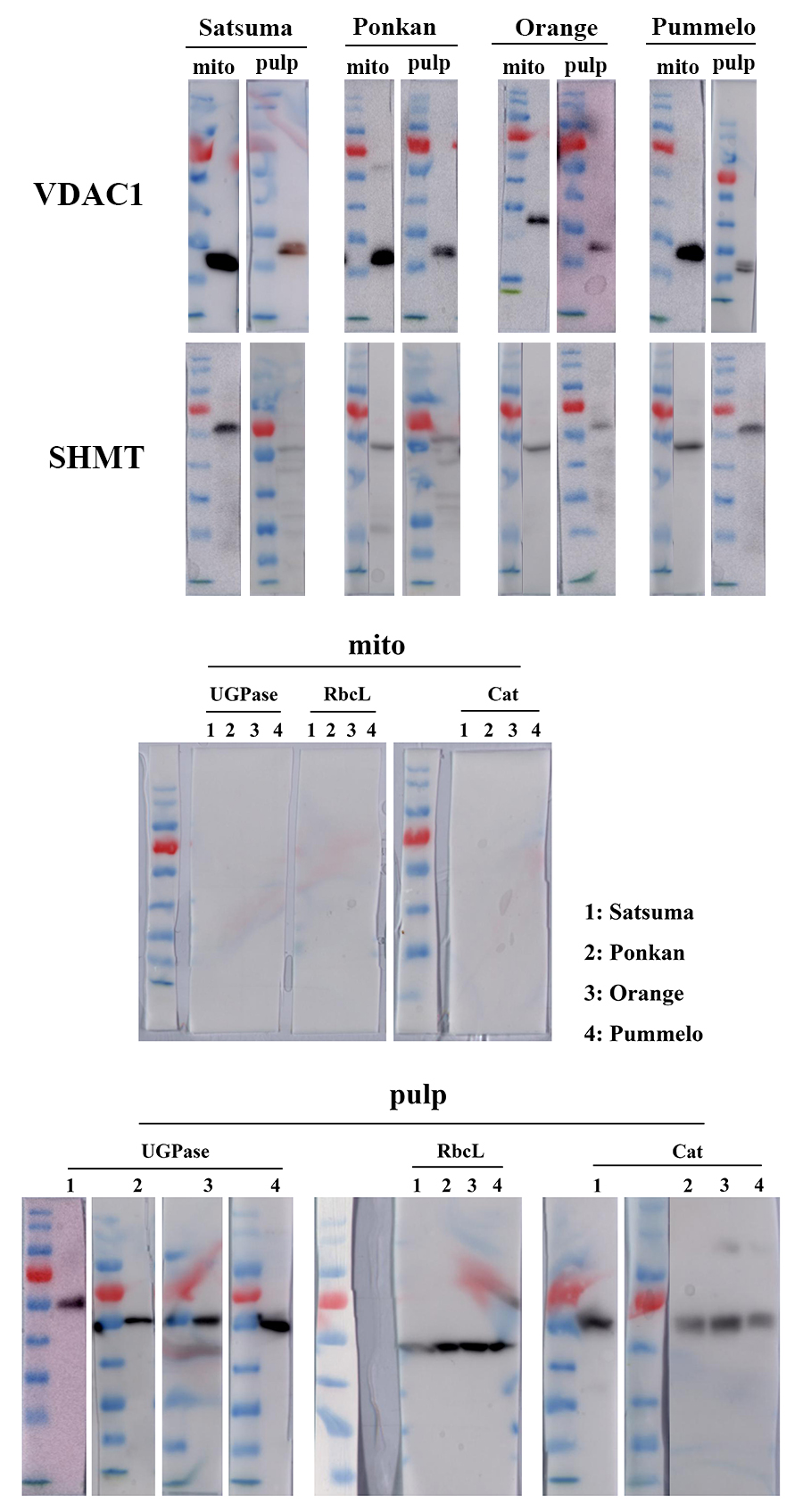


**Figure S6. Uncropped blot images shown in Fig. 2a.** A total of 10 μg of mitochondrial proteins and pulp proteins were separated on 10% SDS-PAGE and blotted onto PVDF membrane. Blots were performed using different marker antibodies against VDAC1 (mitochondrial outer membrane), SHMT (mitochondrial matrix), UGPase (cytoplasm), RbcL (plastid) and Cat (peroxisome).

**Table S1. Summary information about protein identification**

| **Sample** | ***Citrus unshiu*** | | |  | ***Citrus reticulata*** | | |  | ***Citrus sinensis*** | | |  | ***Citrus grandis*** | | | **Total** |
| --- | --- | --- | --- | --- | --- | --- | --- | --- | --- | --- | --- | --- | --- | --- | --- | --- |
|  | **replicate 1** | **replicate 2** | **replicate 3** |  | **replicate 1** | **replicate 2** | **replicate 3** |  | **replicate 1** | **replicate 2** | **replicate 3** |  | **replicate 1** | **replicate 2** | **replicate 3** |  |
| Total spectra | 73479 | 73067 | 73884 |  | 74179 | 72384 | 72314 |  | 70294 | 70817 | 69478 |  | 70987 | 73124 | 71339 | 865346 |
| Spectra (PSM) | 28537 | 26687 | 26724 |  | 26040 | 26938 | 26270 |  | 26646 | 25984 | 25798 |  | 26103 | 27783 | 26314 | 319824 |
| Peptides | 18084 | 16616 | 16747 |  | 16526 | 16491 | 16114 |  | 15611 | 14305 | 13879 |  | 13902 | 15677 | 14315 | 26740 |
| Protein groups | 2807 | 2622 | 2675 |  | 2642 | 2575 | 2572 |  | 2511 | 2294 | 2217 |  | 2200 | 2433 | 2261 | 3755 |
| Total protein groups | 2708 | | |  | 2599 | | |  | 2338 | | |  | 2287 | | | 3353 |

**Table S4. Comparison of mitochondrial proteomes with different species combinations**

| **Species combination** | **No. of overlap** | **overlap rate** |
| --- | --- | --- |
| Satsuma/Ponkan | 2324 | 77.9% |
| Satsuma/Orange | 2034 | 67.5% |
| Satsuma/Pummelo | 1875 | 60.1% |
| Ponkan/Orange | 2033 | 70% |
| Ponkan/Pummelo | 1848 | 60.8% |
| Orange/Pummelo | 1886 | 68.9% |
| Satsuma/Ponkan/Orange | 1906 | 60.3% |
| Satsuma/Ponkan/Pummelo | 1737 | 52.9% |
| Satsuma/Orange/Pummelo | 1704 | 52.6% |
| Ponkan/Orange/Pummelo | 1688 | 53.7% |
| Satsuma/Ponkan/Orange/Pummelo | 1614 | 48.1% |

**Table S6. List of selected candidate proteins for validation by subcellular localization in tobacco leaf**

| **Protein ID** | **Annotation** | **KEGG pathway** | **Arabidopsis ortholog** | **Localization** |
| --- | --- | --- | --- | --- |
| Ciclev10015047m | Malonyl-CoA decarboxylase family protein | Fatty acid metabolism | AT4G04320.2 | mito |
| Cs1g19460.1 | Acyl-coenzyme A thioesterase 13 | Fatty acid metabolism | AT1G04290.1 | partially mito |
| Cs2g03620.1 | 3-oxoacyl-[acyl-carrier-protein] synthase, mitochondrial | Fatty acid metabolism | AT5G46290.1 | partially mito |
| Cs2g15280.1 | Aspartate aminotransferase, mitochondrial | Amino acid metabolism | AT2G30970.2 | mito |
| Cg5g043990.1 | 3-hydroxyisobutyryl-CoA hydrolase-like protein 1, mitochondrial | Amino acid metabolism | AT3G60510.1 | mito |
| Cg9g002610.1 | Lipoamide acyltransferase component of branched-chain alpha-keto acid dehydrogenase complex, mitochondrial | Amino acid metabolism | AT3G06850.2 | mito |
| Cg6g012020.1 | Gamma aminobutyrate transaminase 3, chloroplastic | Amino acid metabolism | AT3G22200.1 | mito |
| Ciclev10007423m | Adenosylmethionine-8-amino-7-oxononanoate transaminases | Biosynthesis of secondary metabolites | AT5G57590.1 | mito |
| Cs9g18510.1 | Folylpolyglutamate synthase | Biosynthesis of secondary metabolites | AT5G41480.1 | mito |
| Cs4g09810.1 | Putative uncharacterized protein | Unknown | - | mito |

**Table S7. Main parameters used for protein identification and quantitative analysis**

| **Item** | **Value** |
| --- | --- |
| Enzyme | Trypsin |
| Max Missed Cleavages | 2 |
| Main search | 6 ppm |
| First search | 20 ppm |
| MS/MS Tolerance | 20 ppm |
| Fixed modifications | Carbamidomethyl (C) |
| Variable modifications | Oxidation (M), Acetyl (Protein N-term) |
| Database pattern | Reverse |
| Peptide FDR | ≤0.01 |
| Protein FDR | ≤0.01 |
| Time window (match between runs) | 2min |
| Protein Quantification | Razor and unique peptides were used for protein quantification. |
| LFQ | True |
| LFQ min. ratio count | 1 |

**Table S9. List of forward and reverse primers used in this study for gene cloning**

| **Protein ID** | **Forward primer (5’-3’)** | **Reverse primer (5’-3’)** |
| --- | --- | --- |
| Ciclev10015047m | ggacagcccagatcaactagtATGAACAAGAGAAGCTTAGCGGTC | gcccttgctcaccatggatccCCTGTATGCATAGTTCACCATGATAC |
| Cs1g19460.1 | ggacagcccagatcaactagtATGGAGTTGGAGTCAGTGAAGAGAT | gcccttgctcaccatggatccCATTTTACTAGAGATAGCAAGGTACTTAGTATG |
| Cs2g03620.1 | ggacagcccagatcaactagtATGCAATCTCTTCAATCCTCTTCTC | gcccttgctcaccatggatccTGGTTTGAATGCAGAAAATGCA |
| Cs2g15280.1 | ggacagcccagatcaactagtATGGCGATGCGGAGCTTG | gcccttgctcaccatggatccAGCAGATTTTGTGACCTCATGAATA |
| Cg5g043990.1 | ggacagcccagatcaactagtATGCAAAGGCTGAAGATTTCAAA | gcccttgctcaccatggatccGTTAAATGCTTCTCGCAGTTTTGT |
| Cg9g002610.1 | ggacagcccagatcaactagtATGATGATCAGTAGAAGGATTTGGC | gcccttgctcaccatggatccTCTCATTTGCAACAGAAGTAACTCAG |
| Cg6g012020.1 | ggacagcccagatcaactagtATGTTAAGAGCAAAAATGGTGACCA | gcccttgctcaccatggatccCTGCTGGGACTTGAGTTCCTTC |
| Ciclev10007423m | ggacagcccagatcaactagtATGCTTCGCCTCCGACGC | gcccttgctcaccatggatccGCAAGACTTCAATTTCTCTTCAACTT |
| Cs9g18510.1 | ggacagcccagatcaactagtATGAAACTCTTAAAGCGTGCTACTCA | gcccttgctcaccatggatccGCTTAACTCATGAAGTGAAGCCAA |
| Cs4g09810.1 | ggacagcccagatcaactagtATGTCGTTAGTGTCGCAAATGAGA | gcccttgctcaccatggatccATCTCCCCTCCCGCGGTA |
