# Supplementary figures and images for: Isolation and comparative proteomic analysis of mitochondria from the pulp of ripening citrus fruit
Source: Hortic Res. 2021 Feb 1;8:31. doi: 10.1038/s41438-021-00470-w (PMC7848011; doi:10.1038/s41438-021-00470-w)

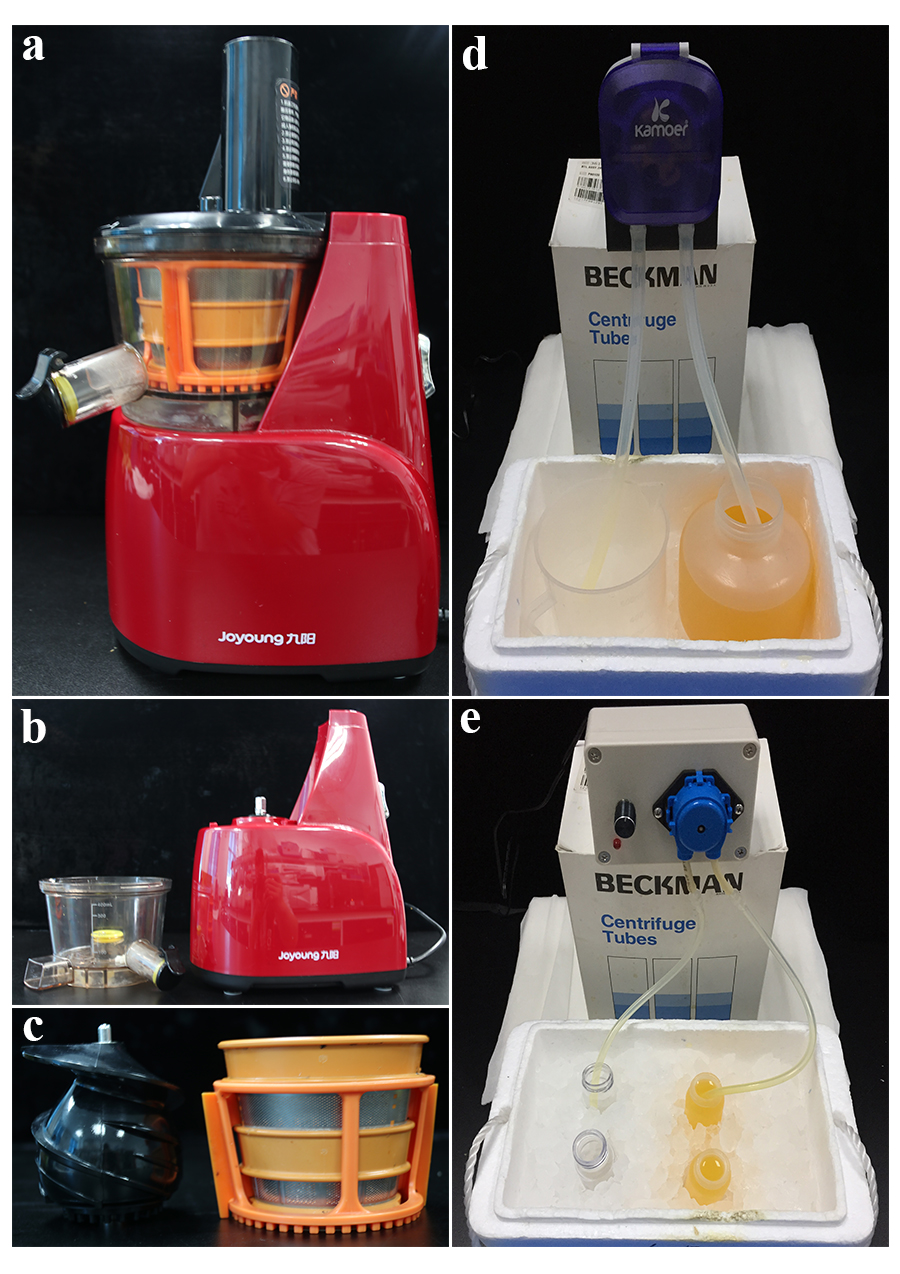

Supplement: Supplementary file 3 — Figure S1. [file 41438_2021_470_MOESM3_ESM.jpg]

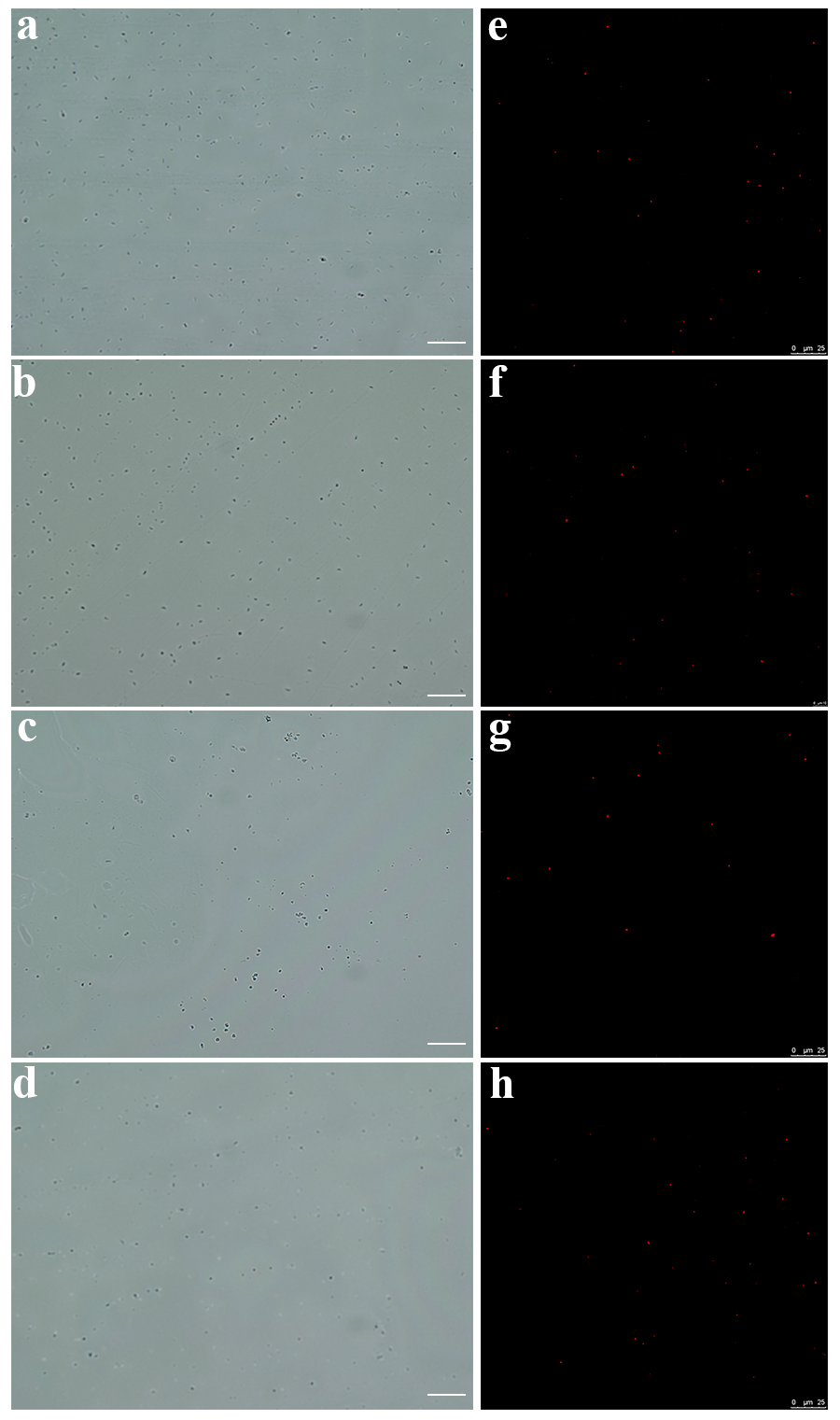

Supplement: Supplementary file 4 — Figure S2. [file 41438_2021_470_MOESM4_ESM.jpg]

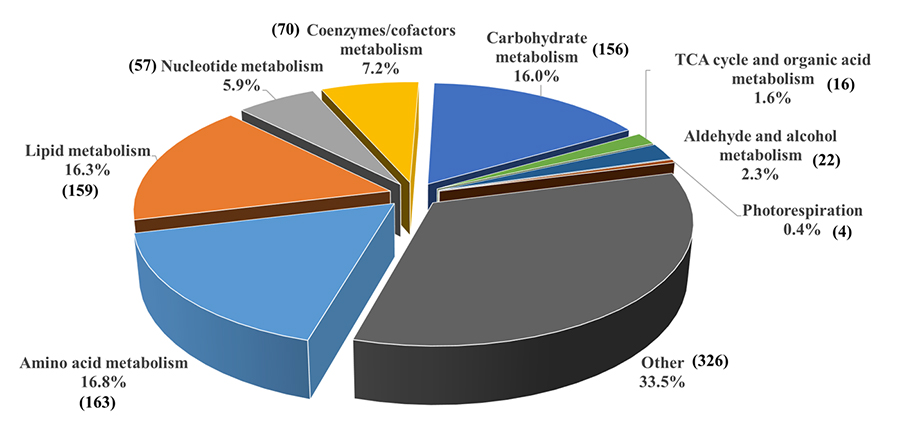

Supplement: Supplementary file 5 — Figure S3. [file 41438_2021_470_MOESM5_ESM.jpg]

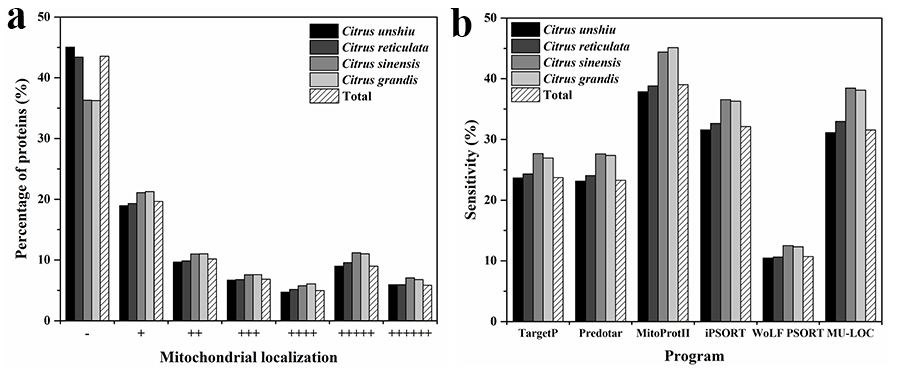

Supplement: Supplementary file 6 — Figure S4. [file 41438_2021_470_MOESM6_ESM.jpg]

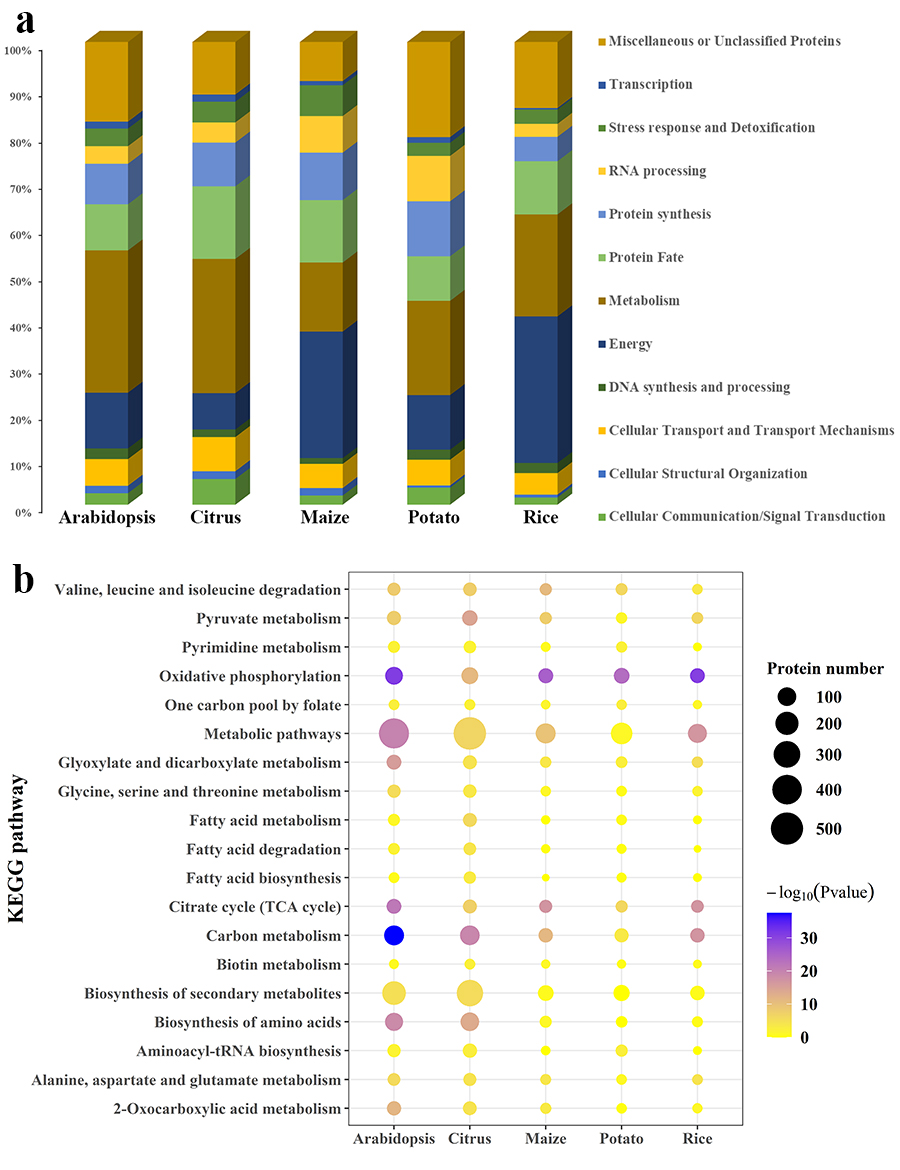

Supplement: Supplementary file 7 — Figure S5. [file 41438_2021_470_MOESM7_ESM.jpg]

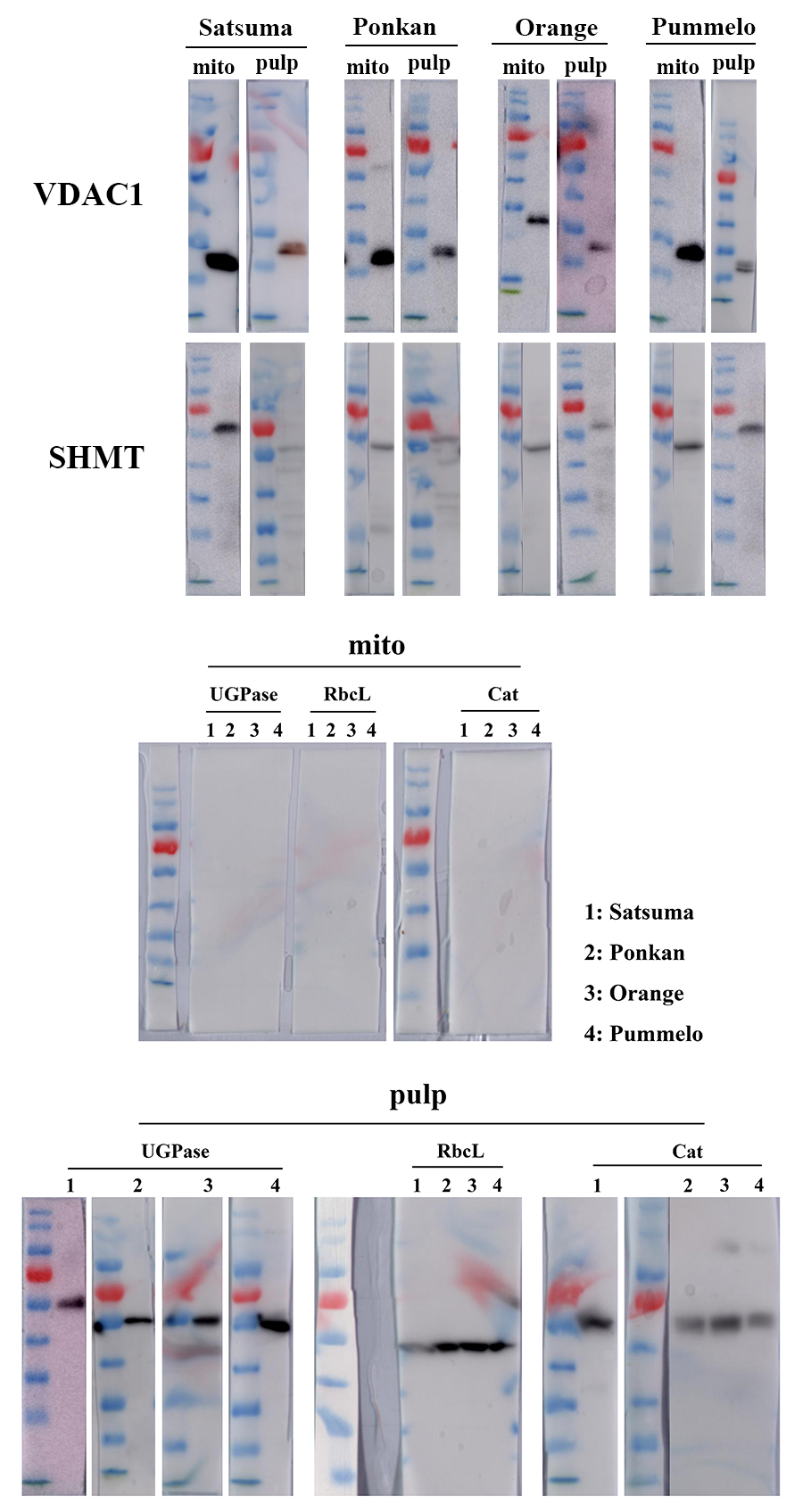

Supplement: Supplementary file 8 — Figure S6. [file 41438_2021_470_MOESM8_ESM.jpg]
